# Artemether-lumefantrine dosing for malaria treatment in young children and pregnant women: A pharmacokinetic-pharmacodynamic meta-analysis

**DOI:** 10.1371/journal.pmed.1002579

**Published:** 2018-06-12

**Authors:** Frank Kloprogge, Lesley Workman, Steffen Borrmann, Mamadou Tékété, Gilbert Lefèvre, Kamal Hamed, Patrice Piola, Johan Ursing, Poul Erik Kofoed, Andreas Mårtensson, Billy Ngasala, Anders Björkman, Michael Ashton, Sofia Friberg Hietala, Francesca Aweeka, Sunil Parikh, Leah Mwai, Timothy M. E. Davis, Harin Karunajeewa, Sam Salman, Francesco Checchi, Carole Fogg, Paul N. Newton, Mayfong Mayxay, Philippe Deloron, Jean François Faucher, François Nosten, Elizabeth A. Ashley, Rose McGready, Michele van Vugt, Stephane Proux, Ric N. Price, Juntra Karbwang, Farkad Ezzet, Rajesh Bakshi, Kasia Stepniewska, Nicholas J. White, Philippe J. Guerin, Karen I. Barnes, Joel Tarning

**Affiliations:** 1 WorldWide Antimalarial Resistance Network, Bangkok, Thailand; 2 Centre for Tropical Medicine and Global Health, University of Oxford, Oxford, United Kingdom; 3 Institute for Global Health, University College London, London, United Kingdom; 4 WorldWide Antimalarial Resistance Network, Cape Town, South Africa; 5 Division of Clinical Pharmacology, Department of Medicine, University of Cape Town, Cape Town, South Africa; 6 Kenya Medical Research Institute–Wellcome Trust Research Programme, Kilifi, Kenya; 7 Institute for Tropical Medicine, Eberhard Karls University of Tübingen, Tübingen, Germany; 8 Malaria Research and Training Center, Department of Epidemiology of Parasitic Diseases, Faculty of Pharmacy, University of Science, Techniques and Technologies of Bamako, Bamako, Mali; 9 Novartis, Basel, Switzerland; 10 Novartis Pharmaceuticals, East Hanover, New Jersey, United States of America; 11 Institut Pasteur du Cambodge, Phnom Penh, Cambodia; 12 Department of Microbiology, Tumor and Cell Biology, Karolinska Institutet, Stockholm, Sweden; 13 Department of Infectious Diseases, Danderyds Hospital, Stockholm, Sweden; 14 Bandim Health Project, Bissau, Guinea-Bissau; 15 Department of Paediatrics, Kolding Hospital, Kolding, Denmark; 16 Department of Women’s and Children’s Health, International Maternal and Child Health, Uppsala University, Uppsala, Sweden; 17 Muhimbili University of Health and Allied Sciences, Dar es Salaam, Tanzania; 18 Karolinska Institutet, Stockholm, Sweden; 19 Department of Pharmacology, University of Gothenburg, Gothenburg, Sweden; 20 Pharmetheus, Uppsala, Sweden; 21 UCSF School of Pharmacy, San Francisco, California, United States of America; 22 Yale School of Public Health, New Haven, Connecticut, United States of America; 23 Institute for Tropical Medicine and Joanna Briggs Institute Affiliate Centre for Evidence Based Health Care Evidence Synthesis and Translation Unit, Afya Research Africa, Nairobi, Kenya; 24 International Development Research Centre, Ottawa, Ontario, Canada; 25 Medical School, Faculty of Health and Medical Sciences, The University of Western Australia, Perth, Western Australia, Australia; 26 Walter and Eliza Hall Institute of Medical Research, Melbourne, Victoria, Australia; 27 Epicentre, Paris, France; 28 Department of Infectious Disease Epidemiology, London School of Hygiene & Tropical Medicine, London, United Kingdom; 29 Faculty of Science, University of Portsmouth, Portsmouth, United Kingdom; 30 Lao–Oxford–Mahosot Hospital–Wellcome Trust Research Unit, Vientiane, Laos; 31 Faculty of Postgraduate Studies, University of Health Sciences, Vientiane, Laos; 32 UMR216 Institut de Recherche pour le Développement, Faculté de Pharmacie, Université Paris Descartes, Paris, France; 33 Centre Hospitalier Universitaire, UMR Inserm 1094 NET, Limoges, France; 34 Shoklo Malaria Research Unit, Mae Sot, Thailand; 35 Myanmar Oxford Clinical Research Unit, Yangon, Myanmar; 36 Amsterdam Medical Centre, Amsterdam, The Netherlands; 37 WorldWide Antimalarial Resistance Network, Darwin, Northern Territory, Australia; 38 Global and Tropical Health Division, Menzies School of Health Research, Darwin, Northern Territory, Australia; 39 Charles Darwin University, Darwin, Northern Territory, Australia; 40 Institute of Tropical Medicine, Nagasaki University, Nagasaki, Japan; 41 WorldWide Antimalarial Resistance Network, Oxford, United Kingdom; 42 Mahidol–Oxford Tropical Medicine Research Unit, Faculty of Tropical Medicine, Mahidol University, Bangkok, Thailand; Burnet Institute, AUSTRALIA

## Abstract

**Background:**

The fixed dose combination of artemether-lumefantrine (AL) is the most widely used treatment for uncomplicated *Plasmodium falciparum* malaria. Relatively lower cure rates and lumefantrine levels have been reported in young children and in pregnant women during their second and third trimester. The aim of this study was to investigate the pharmacokinetic and pharmacodynamic properties of lumefantrine and the pharmacokinetic properties of its metabolite, desbutyl-lumefantrine, in order to inform optimal dosing regimens in all patient populations.

**Methods and findings:**

A search in PubMed, Embase, ClinicalTrials.gov, Google Scholar, conference proceedings, and the WorldWide Antimalarial Resistance Network (WWARN) pharmacology database identified 31 relevant clinical studies published between 1 January 1990 and 31 December 2012, with 4,546 patients in whom lumefantrine concentrations were measured. Under the auspices of WWARN, relevant individual concentration-time data, clinical covariates, and outcome data from 4,122 patients were made available and pooled for the meta-analysis. The developed lumefantrine population pharmacokinetic model was used for dose optimisation through in silico simulations. Venous plasma lumefantrine concentrations 7 days after starting standard AL treatment were 24.2% and 13.4% lower in children weighing <15 kg and 15–25 kg, respectively, and 20.2% lower in pregnant women compared with non-pregnant adults. Lumefantrine exposure decreased with increasing pre-treatment parasitaemia, and the dose limitation on absorption of lumefantrine was substantial. Simulations using the lumefantrine pharmacokinetic model suggest that, in young children and pregnant women beyond the first trimester, lengthening the dose regimen (twice daily for 5 days) and, to a lesser extent, intensifying the frequency of dosing (3 times daily for 3 days) would be more efficacious than using higher individual doses in the current standard treatment regimen (twice daily for 3 days). The model was developed using venous plasma data from patients receiving intact tablets with fat, and evaluations of alternative dosing regimens were consequently only representative for venous plasma after administration of intact tablets with fat. The absence of artemether-dihydroartemisinin data limited the prediction of parasite killing rates and recrudescent infections. Thus, the suggested optimised dosing schedule was based on the pharmacokinetic endpoint of lumefantrine plasma exposure at day 7.

**Conclusions:**

Our findings suggest that revised AL dosing regimens for young children and pregnant women would improve drug exposure but would require longer or more complex schedules. These dosing regimens should be evaluated in prospective clinical studies to determine whether they would improve cure rates, demonstrate adequate safety, and thereby prolong the useful therapeutic life of this valuable antimalarial treatment.

## Abstract 2

Joel Tarning and colleagues investigate the pharmacokinetic and pharmacodynamic properties of artemether-lumefantrine and its principal metabolite in order to inform optimal dosing regimens for treating malaria in all patients, particularly children and pregnant women.

## Author summary

**Why was this study done?:**

- Artemether-lumefantrine (AL) is the most widely used treatment for uncomplicated *Plasmodium falciparum* malaria, and lower cure rates and lumefantrine exposures have been reported in children below 5 years of age and pregnant women.
- Pharmacokinetic-pharmacodynamic trials are generally small, and differences in study designs limit the generalisability of the findings.
- It is crucial to develop an optimised dose regimen that achieves equivalent drug exposure in all patient groups.

**What did the researchers do and find?:**

- Lumefantrine concentration-time data from 4,122 patients from 26 studies were collated and pooled for an individual participant data meta-analysis.
- A population pharmacokinetic-pharmacodynamic model was developed for lumefantrine to understand how body weight, pregnancy, and baseline parasite density influence drug levels.
- Small children and women during their second and third pregnancy trimester displayed lower lumefantrine exposures than non-pregnant adults when receiving the recommended 3-day dosing regimen.
- The developed lumefantrine population pharmacokinetic model was used to evaluate 3 alternative dosing regimens.
- A 5-day regimen of current weight-based standard twice-daily doses for small children and pregnant women beyond their first trimester is most favourable from a pharmacological perspective.

**What do these findings mean?:**

- The proposed AL dosing regimen is expected to provide equivalent lumefantrine exposures safely in all patients as well as to expose one additional asexual life cycle to artemether-dihydroartemisinin.
- The proposed dosing regimen should not only increase cure rates and slow resistance development but also prolong the useful therapeutic life of AL.

## Introduction

Malaria is a major infectious disease in tropical countries, with an estimated 212 (range 148–304) million infections and 429,000 (range 235,000–639,000) deaths in 2015 [1]. Over 90% of the global malaria mortality is reported in sub-Saharan Africa. Children under 5 years of age are the most vulnerable, accounting for 70% of all malaria-related deaths [1]. The World Health Organization recommends that uncomplicated *Plasmodium falciparum* malaria should be treated with an artemisinin-based combination therapy (ACT) [2]. Artemether-lumefantrine (AL) is the most widely used ACT, accounting for 73% of global ACT procurement in 2013 [3], which makes it one of the most widely used anti-infective agents in the world today. Furthermore, AL is well tolerated and safe for the treatment of uncomplicated *P*. *falciparum* (and other malaria species) infections in all age groups. This includes young children and pregnant women in their second and third trimesters, groups with increased morbidity and mortality from falciparum malaria [2]. Sub-optimal drug exposures have been reported following currently recommended doses of AL both in young children and pregnant women in their second and third trimesters [4–12]. On the other hand, similar exposure in pregnant women during their second and third trimester compared to non-pregnant women has also been observed, although the non-pregnant women in this study might not have been as symptomatic as the pregnant women [13]. Moreover, the numbers of patients recruited to these clinical trials were generally small, and differences in study design including selection of comparator therapies and dose regimens and co-administration with fat [14,15] all limit the generalisability of the findings. A meta-analysis could potentially overcome this by pooling individual patient level data from several different studies and characterising both pharmacological properties and the influence of differences in study design, study size, comparator therapies, dose regimens, and inconsistent co-administration with fat. Lumefantrine exposure at day 7 has been evaluated in a meta-analysis previously, but not using a dynamic modelling approach to characterise and quantify pharmacological properties, the influence of covariates, and the relationship between drug concentrations and study outcome [16].

The aim of this study was to assemble a large and therefore sufficiently powered pooled dataset of patients to examine the pharmacokinetic properties of lumefantrine. This approach enabled critical re-evaluation of the current twice-daily 3-day dosing regimen of AL, particularly in children and in pregnant women in their second and third trimesters. As both lumefantrine and its principal metabolite, desbutyl-lumefantrine, have antimalarial activity, we also designed a pharmacokinetic analysis incorporating both compounds.

## Methods

### Model building

Pharmacokinetic data, clinical covariates, and efficacy data from patients treated with AL were used for this individual patient data meta-analysis. A search was conducted in PubMed, Embase, ClinicalTrials.gov, Google Scholar, conference proceedings, and the WorldWide Antimalarial Resistance Network (WWARN) pharmacology publication database to identify relevant antimalarial clinical studies published between 1 January 1990 and 31 December 2012 [16] in which pharmacokinetic parameters as well as clinical covariates in patients treated with AL were recorded. The search strategy used key terms “lumefantrine pharmacokinetics” or “lumefantrine concentration” and “clinical study”.

Under the auspices of WWARN, investigators were invited to participate in this individual patient data meta-analysis. Individual study protocols were available for all trials included, either from the publication or as a metafile submitted with the raw data. Individual patient data from eligible studies were shared, standardised, and collated using a methodology described in the WWARN clinical and pharmacology data management and statistical analysis plans [17,18] and previously published research [16].

Concentration-time data and clinical covariates from patients contributing 2 or more venous plasma samples were used to build the pharmacokinetic models. Patients contributing only 1 pharmacokinetic sample could not be used for model development as between-patient variability could not be dissected from residual variability. Lumefantrine concentration data in different sampling matrices (i.e., venous blood and capillary plasma and blood) and lumefantrine concentration data after administration of different formulations (i.e., crushed tablets and dispersible tablets) were not used for the formal pharmacokinetic model development due to the sparse sampling schedules (i.e., <2 samples per patient for crushed tablets, dispersible tablets, venous blood, and capillary blood) and to avoid introducing additional sources of variability (i.e., capillary plasma samples). Consequently, lumefantrine concentration-time data in venous blood, capillary blood, and capillary plasma as well as lumefantrine concentration-time data from dispersible tablets and crushed tablets were evaluated using a post hoc correction factor at residual variability level with all other pharmacokinetic parameters fixed. Patients contributing <2 venous plasma samples were used for external validation of the developed population pharmacokinetic models.

Both a lumefantrine population pharmacokinetic model and a separate lumefantrine/desbutyl-lumefantrine population pharmacokinetic drug-metabolite model were developed. The natural logarithms of the concentration data were modelled in NONMEM v.7.3 (ICON Development Solutions, Ellicott City, MD) on a Windows XP operating system (Microsoft, Seattle, WA) with a G95 Fortran compiler (Free Software Foundation, Boston, MA).

The developed lumefantrine population pharmacokinetic model was used to generate individual post hoc pharmacokinetic parameter estimates. The resulting lumefantrine concentration-time profiles were subsequently used to link lumefantrine plasma concentrations and clinical study outcome (i.e., cure defined as absence of PCR-corrected recrudescent infection during follow-up) using a time-to-event approach (pharmacokinetic-pharmacodynamic time-to-event model). All outcome data later than day 42 were censored at day 42, and novel infections were censored. The lumefantrine/desbutyl-lumefantrine drug-metabolite model was not combined with the time-to-event approach due to the small sample size relative to the lumefantrine population pharmacokinetic model.

More technical information regarding the pharmacokinetic model building process and the pharmacokinetic-pharmacodynamic time-to-event model building process can be found in S1 Text.

### Dose optimisation simulations

The developed lumefantrine pharmacokinetic model was subsequently used for in silico evaluation and comparison of 3 alternative dosing regimens, with plasma lumefantrine concentration on day 7 selected as the pharmacokinetic endpoint [19,20]. This pharmacokinetic endpoint has been used as a target for AL treatment as it correlates with cure rate. The day 7 concentration reflects lumefantrine exposure over the previous 3 asexual cycles (i.e., 6 days for *P*. *falciparum*) [19,20]. Plasma or whole blood [21] lumefantrine concentration on day 7 therefore constitutes a clinically relevant and practical surrogate of overall drug exposure, with suggested target day 7 lumefantrine concentrations of 175 ng/ml, 200 ng/ml, and 280 ng/ml in different studies [12,16,22,23].

A variety of alternative dosing regimens for young children and pregnant women were simulated, and lumefantrine pharmacokinetic parameters were compared to those following administration of the standard dose regimen in non-pregnant adults. Alternative dosing regimens included an increased dosage (1 extra tablet containing 20 mg artemether and 120 mg lumefantrine added to current weight-based standard dose at each twice-daily dose for 3 days), an extended treatment (5-day regimen of current weight-based standard twice-daily doses), and an intensified treatment (current weight-based standard dosage administered 3 times daily for 3 days). More technical information regarding the in silico dose optimisation simulations can be found in S1 Text.

### Ethical approval

All data included in this analysis were obtained in accordance with the laws and ethical approvals applicable in the countries in which the studies were conducted, and were from clinical studies in which blood samples were obtained with the knowledge and consent of the individuals to which they relate. Data were fully anonymised either before or during the process of uploading to the WWARN pharmacology database. Ethical approval to conduct individual participant data pooled analyses was granted to WWARN by the Oxford Tropical Research Ethics Committee (OxTREC).

## Results

**Fig 1:**
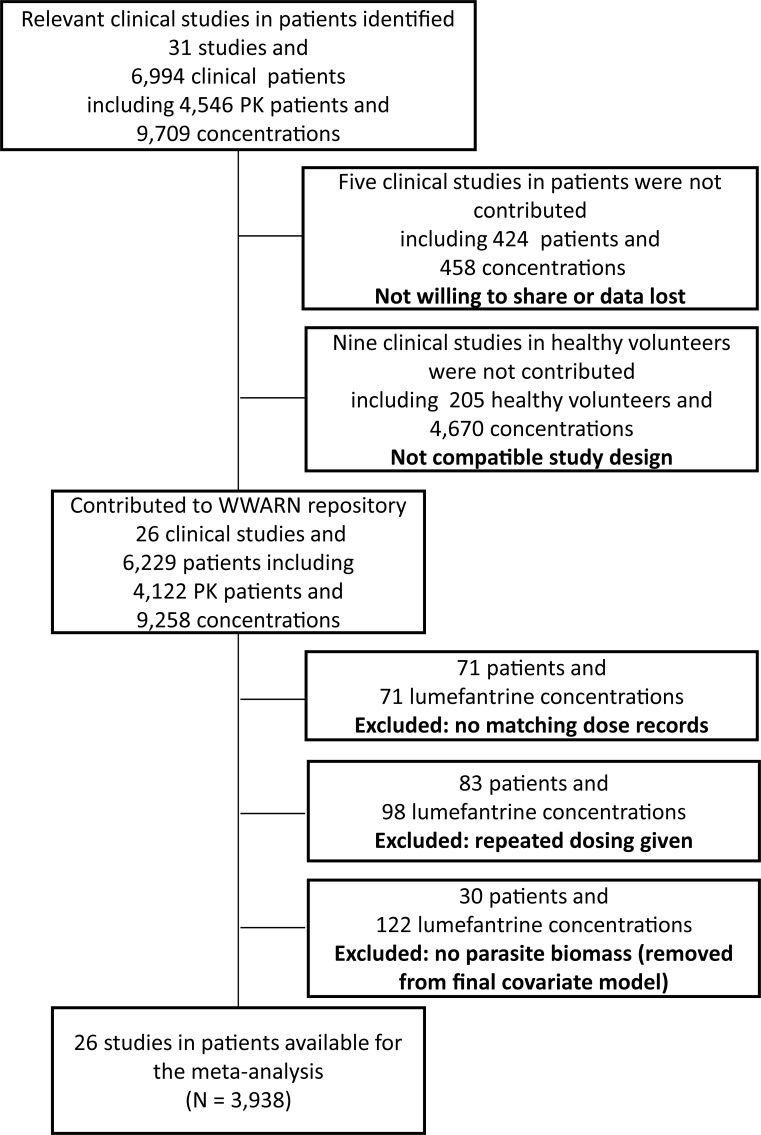
Patient data disposition. PK, pharmacokinetic; WWARN, WorldWide antimalarial resistance Network.

Lumefantrine concentration-time data from 4,122 patients from 26 studies were uploaded to the WWARN pharmacology database (Fig 1; S1 Table). These data were categorised into 3 different geographic areas (Africa, Oceania, and Southeast Asia) comprising 12 countries (Benin, Guinea-Bissau, Tanzania, Uganda, Kenya, Mali, Mozambique, Liberia, Papua New Guinea, Lao People’s Democratic Republic [Laos], Thailand, and Cambodia). Lumefantrine concentrations were available for analysis in 4 different matrices: 2,312 patients contributed venous plasma samples, 595 patients contributed venous whole blood samples, 191 patients contributed capillary plasma samples, and 840 patients contributed capillary whole blood samples. In total, 154 patients were excluded from the analysis, 71 because of missing dosing information and 83 who took repeated dosing (i.e., retreatment). A further 30 patients were excluded from the evaluation of pre-treatment parasitaemia as a covariate because relevant parasitological data were missing. Venous plasma data from 1,347 out of 2,312 patients who contributed at least 2 samples was used for the development of the lumefantrine population pharmacokinetic model. The remaining 400, 278, and 287 patients contributed only 1 venous plasma sample per patient after treatment with intact, crushed, and dispersible tables, respectively, and were therefore only used for external validation and evaluation of formulation effects in a separate analysis (Table 1). Approximately 37% of the patients were children below 10 years of age and 3.12% were pregnant women (median [range: interquartile range] 23.0 [13.1–38.0: 19.1–30.0] weeks gestational age). Data from 3,486 patients were available for the development of the pharmacokinetic-pharmacodynamic time-to-event model. Approximately 59% of the patients were below 10 years of age and 4.7% were pregnant women (median [range: interquartile range] 22.0 [13.1–39.0: 18.5–28.0] weeks gestational age). Venous plasma data from 159 patients were used for the development of the simultaneous lumefantrine/desbutyl-lumefantrine population pharmacokinetic model. Approximately 57% of the patients were below 10 years of age and 8.81% were pregnant women (median [range: interquartile range] 23.4 [16.2–38.0: 20.8–30.4] weeks gestational age).

**Table 1:** Demographic summary of the data used for the pharmacokinetic and pharmacokinetic-pharmacodynamic models.

Model	Data	Studies	Study sites	Study size	Dose regimen	Dose (mg/kg)	Sampling matrix	Sample size LF (>LLOQ)	Samples/patient LF	Sample size DLF (>LLOQ)	Samples/patient DLF	Male/female (%)	Age (years)	Body weight (kg)	*z*-Score (weight for age)	Admission parasitaemia (μl^−1^)	Pregnant women (%)	Estimated gestational age (weeks)
**PK LF model data**	Model building data	[4,9,14,22,24–32]	3, 4, 8, 9, 10, 12, 17, 19	1,347	6 over 3 days, 6 over 5 days, 4 over 3 days, 3 over 3 days	10.2 [3.2–20.9: 8.9–12.0]	Venous plasma	5,949	3 [2–26: 2–5]	—	—	55.9/44.1	16.6 [0.5–78.0: 6.1–26.9]	42 [6–150: 17–52]	−1.2 [−5.3 to 2.6: −2.0 to −0.5]	9,450 [13–450,000: 2,240–38,700]	3.1	23 [13–38: 19–30]
	Intact tablets validation data	[27,28,30,32–36]	7, 9, 12, 15, 17, 20, 19	400	6 over 3 days, 6 over 5 days, 4 over 3 days, 3 over 3 days	10.0 [6.3–18.8: 8.9–12.0]	Venous plasma	394	1 [1–1: 1–1]	—	—	65.5/34.5	8.0 [0.5–80: 3.0–23.0]	21 [6–72: 12–50]	−1.3 [−5.0 to 3.5: −2.0 to −0.7]	27,300 [21–422,000: 7,540–62,000]	0.3	—
	Crushed tablets data	[37]	1, 5, 11, 13, 21	278	6 over 3 days	12.0 [8.2–20.0: 10.0–13.8]	Venous plasma	264	1 [1–1: 1–1]	—	—	51.8/48.2	3.4 [0.3–12.4: 2.3–5.6]	13 [6–34: 11–17]	−0.9 [−4.2 to 2.7: −1.6 to −0.4]	30,600 [2,040–629,000: 9,130–72,200]	0	—
	Dispersible tablets data	[37]	5, 11, 13, 1, 21	287	6 over 3 days	12.0 [8.2–21.8: 10.0–14.1]	Venous plasma	275	1 [1–1: 1–1]	—	—	49.8/50.2	3.5 [0.0–12.4: 2.2–5.5]	14 [6–34: 11–17]	−1.0 [−3.8 to 10.5: −1.7 to −0.1]	25,000 [520–197,000: 11,500–60,100]	0	—
	Venous blood data	[38,39]	14	595	6 over 3 days	10.9 [5.9–18.9: 9.4–12.6]	Venous blood	540	1 [1–1: 1–1]	—	—	50.9/49.1	11.0 [1.0–86.7: 6.6–16.2]	28 [7–82: 19–47]	−1.1 [−3.2 to 5.7: −1.7 to −0.3]	976 [16–285,000: 168–7,380]	0	—
	Capillary plasma data	[6,29]	12, 17	191	6 over 3 days	9.2 [5.8–13.7: 8.1–10.0]	Capillary plasma	895	5 [1–7: 4–5]	—	—	0/100	23.0 [15.0–42.0: 19.0–27.5]	52 [35–83: 48–59]	—	3,180 [24–194,000: 551–16,000]	100	23 [13–39: 19–29]
	Capillary blood data	[40–43]	2, 3, 6, 16, 18, 22	840	6 over 3 days	12.0 [8.1–24.0: 10.0–14.1]	Capillary blood	776	1 [1–1: 1–1]	—	—	49.8/50.2	3.0 [0.3–14.6: 1.9–4.3]	12 [5–51: 10–15]	−0.9 [−5.3 to 4.5: −1.7 to −0.1]	35,600 [140–524,000: 9,330–74,400]	0	—
**PK LF/DLF model data**	Model building data	[4,9,26]	8, 10, 17	159	6 over 3 days	11.2 [3.2–18.5: 9.9–12.9]	Venous plasma	832	4 [2–16: 3–4]	735	3 [2–16: 3–3]	41.5/58.5	10.0 [1.0–78.0: 4.6–17.5]	21 [7–150: 14–44]	−1.7 [−4.1 to 1.1: −2.1 to −1.1]	12,600 [91–4 × 10^5^: 2,240–51,800]	8.8	23 [16–38: 21–30]
	Validation data	[4,26,27]	8, 9, 17	135	6 over 3 days	10.0 [5.3–13.3: 8.6–11.6]	Venous plasma	263	2 [1–15: 1–2]	127	1 [1–1: 1–1]	54.1/45.9	3.0 [0.5–35.0: 1.9–4.0]	11 [6–49: 10–14]	−1.5 [−5.3 to 2.6: −2.3 to −0.9]	36,600 [280–450,000: 11,600–101,000]	1.5	18 [13–22: 15–20]
**PK/PD day 42 efficacy data**	All data	[4,6,9,14,22,25–43]	1, 2, 3, 4, 5, 6, 7, 8, 9, 10, 11, 12, 13, 14, 15, 16, 17, 18, 19, 20, 21, 22	3,486	6 over 3 days, 6 over 5 days, 4 over 3 days, 3 over 3 days	10.8 [3.2–24.0: 9.2–12.6]	Venous plasma, capillary plasma, venous blood, capillary blood	—	—	—	—	52.6/47.4	6.6 [0.0–86.7: 3.1–19.0]	19 [5–150: 12–46]	−1.0 [−5.3 to 10.5: −1.8 to −0.3]	16,400 [13–629,000: 2,730–50,300]	4.7	22 [13–39: 19–28]
	African children <15 kg	[26,28,36,37,39–43]	1, 2, 3, 5, 6, 7, 8, 11, 12, 13, 14, 16, 18, 21, 22	1,210	6 over 3 days	10.7 [8.1–24.0: 9.2–12.0]	Venous plasma, venous blood, capillary blood	—	—	—	—	50.3/49.7	2.5 [0.0–14.5: 1.7–3.4]	11 [5–15: 10–13]	−1.1 [−5.3 to 10.5: −1.9 to −0.5]	35,500 [120–629,000: 11,400–72,800]	0	—
	African children 15–25 kg	[25,26,28,36–43]	1, 2, 3, 4, 5, 6, 7, 8, 11, 12, 13, 14, 16, 18, 21, 22	638	6 over 3 days	13.3 [9.8–18.9: 12.0–15.0]	Venous plasma, venous blood, capillary blood	—	—	—	—	50/50	5.7 [2.1–18.8: 4.4–7.5]	18 [15–25: 16–20]	−0.7 [−4.1 to 2.3: −1.4 to 0.0]	17,600 [32–4 × 10^5^: 3,530–48,300]	0	—
	Southeast Asian pregnant women	[4,6]	17	113	6 over 3 days	9.8 [7.4–13.7: 9.1–10.4]	Venous plasma, capillary plasma	—	—	—	—	0/100	24.0 [14.0–42.0: 20.0–33.0]	49 [35–65: 46–53]	—	3,190 [57–154,000: 624–20,100]	100	23 [13–39: 18–291]

Data are presented as median [range: interquartile range].

Included study sites were Benin (1); Bandim Health Projects study area (Bandim, Belem, and Cuntum), Guinea-Bissau (2); Fukayosi, Tanzania (3); Kampala, Uganda (4); Kenya (5); Kibaha District, Tanzania (6); Kilifi, Kenya (7); Kilombero District, Tanzania (8); Madang and East Sepik Provinces, Papua New Guinea (9); Madang Province, Papua New Guinea (10); Mali (11); Mbarara, Uganda (12); Mozambique, Mozambique (13); Nimba County, Liberia (14); Phalanxay District, Laos (15); Sekou, Liberia (16); Shoklo Malaria Research Unit, Thailand (17); Allada, Benin (18); Bangkok, Thailand (19); Battambang Province, Cambodia (20); Tanzania (21); and Yombo, Tanzania (22).

DLF, desbutyl-lumefantrine; LF, lumefantrine; LLOQ, lower limit of quantification; PD, pharmacodynamic; PK, pharmacokinetic.

The following covariates were selected prospectively and their influence evaluated using a population modelling approach: body weight, pregnancy, estimated gestational age, baseline parasitaemia, dosage (mg/kg), dose per occasion (mg), total daily dose (mg), total dose (mg), and age-for-weight *z-*score. Some clinically relevant covariates were not available for all patients; these included haemoglobin (available for 2,901 patients; either directly measured or calculated from measured haematocrit [44]) and baseline body temperature (axillary available for 3,029 patients, tympanic available for 427 patients, and oral available for 100 patients). Pooling of temperature data (i.e., axillary, oral, and tympanic) was considered unreliable due to potential discrepancies between study-site procedures and was therefore not performed. A separated covariate analysis was performed on a subset of data in order to evaluate baseline body temperature and haemoglobin as covariates.

### Lumefantrine pharmacokinetic model

#### Structural model

**Fig 2:**
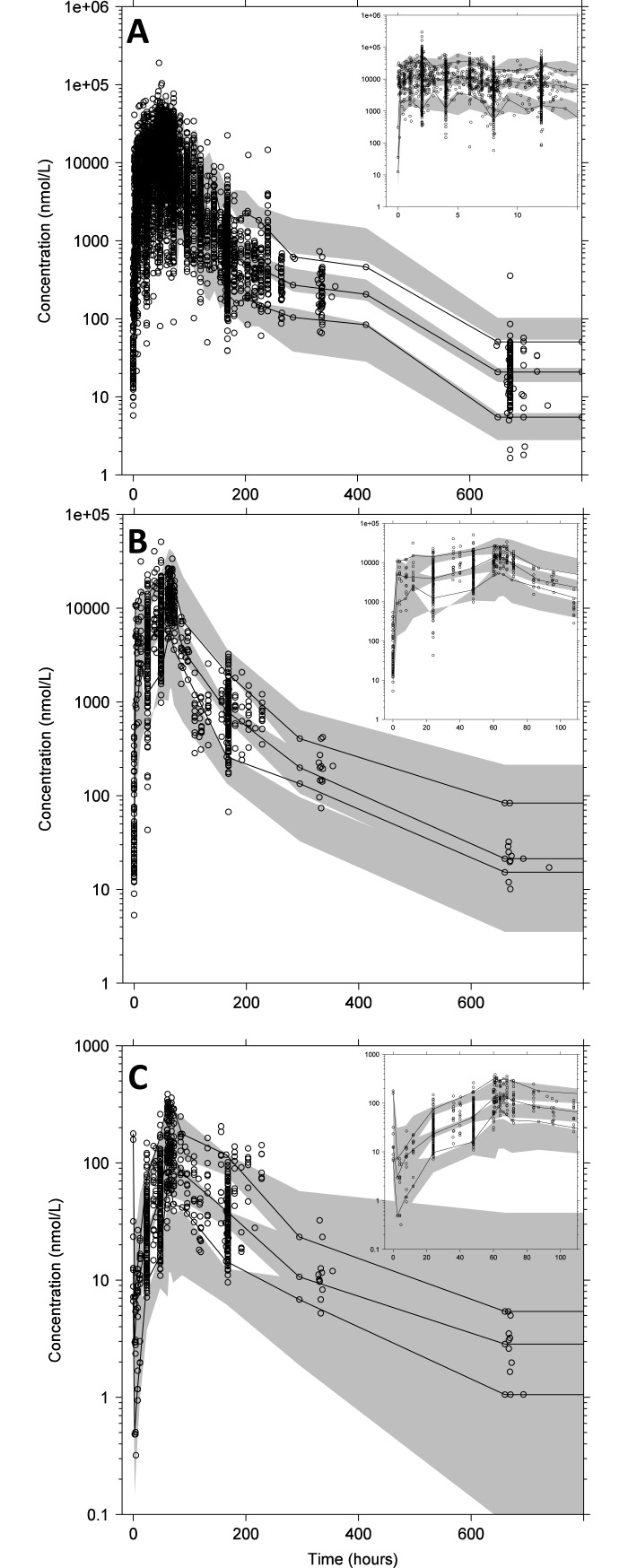
Prediction-corrected visual predictive checks. (A) Prediction-corrected visual predictive check of the lumefantrine population pharmacokinetic model, with the insert representing the first 15 hours after dose. (B and C) Prediction-corrected visual predictive check of the lumefantrine/desbutyl-lumefantrine drug-metabolite model stratified for lumefantrine (B) and desbutyl-lumefantrine (C). The inserts in (B) and (C) show the predictive performance during the first 110 hours after dose. Open circles represent observed plasma concentration data. The solid lines represent the 5th, 50th, and 95th percentiles of the observed data. The grey shaded areas represent the 95% confidence intervals of the simulated (*n =* 2,000) percentiles.

The lumefantrine pharmacokinetic model was developed based on data from 1,347 patients. This subset contained relatively densely sampled venous plasma data (i.e., 2 or more samples per patient) and covered a wide range of covariates (Table 1). Disposition pharmacokinetics was best described using a 2-compartment model, as this model was superior to a 1-compartment model (*p* < 0.001; Δ−2LL = −2,361; ΔAIC = −2,357). The addition of a third compartment did not result in further significant improvement (*p* > 0.01; Δ−2LL = −6.97, ΔAIC = −2.97). A first-order absorption model described the lumefantrine absorption characteristics adequately, and residual variability was described using an additive error model on logarithmic data (Fig 2).

#### Disease- and dosage-related covariate model

Day 7 lumefantrine concentrations decreased with increasing pre-treatment parasitaemias described using a power relationship between bioavailability and pre-treatment parasite count ($F=\theta (n)\times e^{\theta_{parasitaemia}-(parasitaemia-4.2)}$; coefficient −0.643; *p <* 0.001; Δ−2LL = −49.4; ΔAIC = −47.4; Table 2). Moreover, lumefantrine displayed dose-limited absorption, which was parameterised in the model using a maturation effect (50% saturation of the absorption [dose_50_] at 3.42 mg/kg; dose_90_ at approximately 36 mg/kg) on relative bioavailability (*p <* 0.001; Δ−2LL = −64.6; ΔAIC = −62.6; Table 2).

**Table 2:** Population parameter estimates from the final lumefantrine pharmacokinetic model.

Parameter	Fixed effects		Random effects	
	Population estimate (BS estimate)	%RSE (95% CI)	%CV for IIV (BS estimate)	%RSE (95% CI)
*F*	1 (fixed)	—	70.3 (70.2)	5.95 (65.3–75.3)
Box–Cox shape parameter on *F*	−0.343 (−0.342)	19.5 (−0.469 to 0.215)	—	—
*k*_a_ (h^−1^)	0.0386 (0.0388)	2.72 (0.0368 to 0.0410)	—	—
CL/*F* (l/h)	1.35 (1.36)	29.7 (0.538 to 2.19)	—	—
*V*_C_/*F* (l)	11.2 (11.6)	30.3 (4.65 to 18.8)	144 (141)	10.8 (115–165)
*Q/F* (l/h)	0.344 (0.350)	29.8 (0.137 to 0.566)	—	—
*V*_P_/*F* (l)	59.0 (59.5)	29.7 (23.6 to 96.4)	—	—
Dose_50_ (mg/kg) on *F*	3.86 (4.07)	41.5 (1.25 to 8.04)	—	—
Pregnancy on *k*_a_	0.352 (0.355)	21.2 (0.212 to 0.510)	—	—
Parasitaemia on *F*	−0.643 (−0.635)	13.0 (−0.793 to 0.473)	—	—
σ	0.323 (0.323)	4.88 (0.293 to 0.357)	—	—

Coefficient of variation (%CV) for inter-individual variability (IIV) was calculated as $100\times \sqrt{e^{estimate}-1}$. The relative standard error (%RSE) was calculated as $100\times \frac{Standard\;deviation}{Average\;parameter\;estimate}$ from 1,000 iterations of a non-parametric bootstrap (BS) procedure. The 95% confidence interval (95% CI) is displayed as the 2.5th to 97.5th percentile of the BS estimate, and the BS estimate as the average value.

*F*: relative bioavailability; Box–Cox shape parameter: shape parameter on Box–Cox transformation; *k*_a_: absorption rate constant; CL/*F*: elimination clearance; *V*_C_/*F*: apparent central volume of distribution; *Q/F*: inter-compartmental clearance; *V*_P_/*F*: apparent peripheral volume of distribution; dose_50_: dose (mg/kg) needed for half of maximum dose-dependent saturation of *F*; pregnancy: categorical covariate effect of pregnancy on *k*_a_; parasitaemia: continuous covariate effect of enrolment parasite density on *F*; and σ: additive residual error on log scale. All parameters were centred on a non-pregnant patient weighing 42 kg with an admission parasitaemia of 15,800 parasites/μl.

Clearance and volume parameters were centred on the median body weight (WT) and scaled allometrically ($CL\;and\;Q=\theta (n)\times {\frac{WT}{42}}^{\frac{3}{4}},\;V=\theta (n)\times \frac{WT}{42}$); dose-dependent absorption was implemented as a saturation model ($F=\theta (n)\times (1-\frac{Dosage}{\theta_{{Dosage}_{50}}+Dosage})$); baseline parasitaemia was implemented as an exponential relationship centred on the median natural logarithm transformed value ($F=\theta (n)\times (e^{\theta_{parasitaemia}-(parasitaemia-4.2)})$), and the categorical pregnancy effect was implemented as a proportional effect (*k*_a_ = θ(*n*) × (1 + θ_pregnancy_)).

#### Children

**Fig 3:**
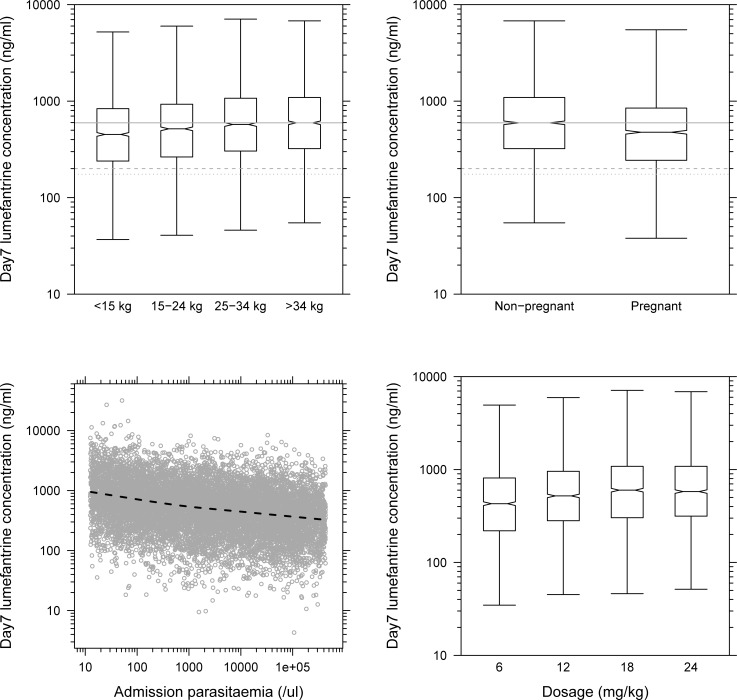
Body weight, pregnancy status, admission parasitaemia, and dosage effects on predicted day 7 venous plasma lumefantrine concentrations (*n =* 2,000). Body weight (top left); pregnancy status (top right); admission parasitaemia (bottom left); dosage (bottom right). Boxes and whiskers represent 25%–75% and 2.5%–97.5% of the data, respectively. The grey solid, dashed, and dotted lines represent 596 ng/ml (median lumefantrine concentration at day 7 in non-pregnant adult patients after a standard treatment), 200 ng/ml [16], and 175 ng/ml [23], respectively. The dotted black line in the parasitaemia panel represents the mean of the simulated data (open grey circles).

The currently recommended 6-dose regimen in children weighing <15 kg and 15–24 kg resulted in 24.2% and 13.4% lower predicted median venous lumefantrine concentrations at day 7, respectively, when compared to adult patients (Fig 3). This resulted from body weight, implemented as a covariate on clearance ($CL\;and\;Q=\theta (n)\times {\frac{WT}{42}}^{\frac{3}{4}}$) and volume ($V=\theta (n)\times \frac{WT}{42}$) parameters (*p <* 0.001; Δ−2LL = −271; ΔAIC = −271; Table 2).

Furthermore, weight-for-age *z-*scores [45] were calculated and tested as a linear covariate for children below 5 years of age (*n* = 281) or below 3 years of age (*n* = 139). Weight-for-age *z-*scores did not correlate with pharmacokinetic parameters in any of the tested age ranges, and age as a proxy for hepatic enzyme maturation did not significantly improve the model fit when included as a covariate on elimination clearance.

#### Pregnant women

Lumefantrine distribution kinetics was substantially affected in pregnant women during their second and third trimester when compared to non-pregnant adults (*p <* 0.001; Δ−2LL = −21.8; ΔAIC = −19.8; Table 2), resulting in 20.2% lower day 7 venous lumefantrine concentrations (*k*_a_ = θ(*n*) × (1 + θ_pregnancy_)).

### Baseline body temperature and haemoglobin

Haemoglobin and admission body temperature were not formally evaluated using a stepwise modelling approach in the population pharmacokinetic model as these covariates were missing for more than 20% of the patients. However, the covariates were evaluated in a separate sub-group covariate analysis, and baseline body temperature did not correlate with any lumefantrine pharmacokinetic parameter in the current population pharmacokinetic model. Measured or haematocrit-derived haemoglobin concentration was available for 74% of the patients and correlated significantly with lumefantrine inter-compartmental clearance (*p <* 0.001; Δ−2LL = −19.3; ΔAIC = −17.3; exponential relationship with an exponent of 0.0629) and apparent peripheral distribution volume (*p <* 0.001; Δ−2LL = −11.5; ΔAIC = −9.5; power relationship with a power of 1.44). This correlation resulted in increasing day 7 venous plasma lumefantrine concentrations with higher haemoglobin levels.

#### Model validation

The final model showed accurate and precise predictive power without indications of model misspecification, using internal data as well as external validation data from 401 patients with sparse venous plasma sampling (Table 1; Figs 2, S1 and S2). The bootstrap diagnostics (*n* = 1,000) confirmed robust parameter estimates with reasonable relative standard errors (Table 2). Eta-shrinkage on apparent volume of distribution of the central compartment and relative bioavailability was 47.5% and 15.2%, respectively.

More technical information regarding the lumefantrine pharmacokinetic model can be found in S2 Text.

#### Matrix and formulation effects

Prediction-corrected visual predictive checks for matrix- and formulation-related subsets of the data were generated from the lumefantrine model on venous plasma data using proportional adjustment and showed adequate predictive performance except for capillary plasma (S2 Fig). Only venous blood data displayed a minor over-prediction of the 5th and 95th percentiles (S2 Fig), although mean trends could still be predicted accurately, and estimated proportional differences in lumefantrine exposures would therefore be unbiased. Lumefantrine concentrations were on average 11.3%, 18.3%, and 23.8% lower in studies measuring venous blood (*n =* 595), capillary plasma (*n =* 191), and capillary blood (*n =* 840), respectively, when compared to the studies measuring lumefantrine in venous plasma after intact tablets (Table 1; S2 Fig). Venous plasma lumefantrine concentrations in studies where patients received dispersible (*n =* 287) and crushed (*n =* 278) tablets were on average 6.31% and 26.0% higher, respectively, when compared to studies measuring lumefantrine in venous plasma after intact tablets (Table 1; S2 Fig).

### Pharmacokinetic-pharmacodynamic time-to-event model

**Fig 4:**
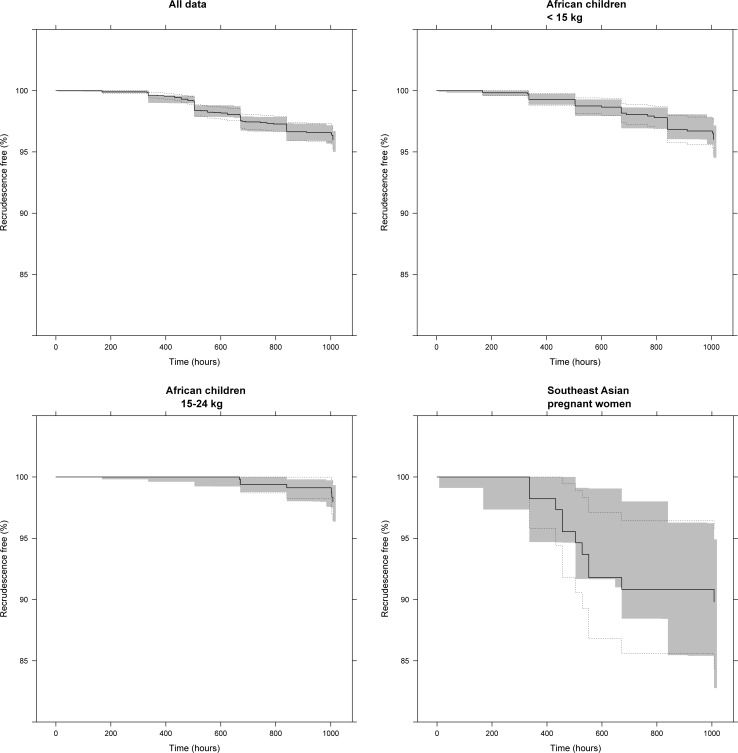
Visual predictive check of the pharmacokinetic-pharmacodynamic time-to-event model. The grey shaded area represents the 95% confidence interval of the simulated (*n =* 2,000) data. Solid and dashed black lines represent the Kaplan–Meier estimator and corresponding standard errors. Panels show all data (top left), African children <15 kg (top right), African children ≥15 kg and <25 kg (bottom left), and pregnant women from Southeast Asia (bottom right).

For the pharmacokinetic-pharmacodynamic time-to-event model, individual pharmacokinetic parameter estimates from 3,486 patients were fixed and evaluated with the treatment outcome at day 42 (Table 3). Overall, 93 out of 3,486 patients (2.67%) had recrudescent infections. A Gompertz hazard model with a sigmoidal *E*_MAX_ lumefantrine drug effect model provided accurate predictive power in the visual predictive check (Fig 4), but parameter estimates lacked precision and accuracy (Table 3). No statistically significant covariates were found for the pharmacodynamic parameters in the full dataset, which could be a consequence of biased distribution of covariates over the geographical regions and the small number of recrudescent infections.

**Table 3:** Parameter estimates from the pharmacokinetic-pharmacodynamic time-to-event model.

Parameter	Day 42 study outcome							
	All data		African children <15 kg		African children 15–25 kg		Southeast Asian pregnant women	
	Population estimate (BS estimate)	%RSE (95% CI)	Population estimate (BS estimate)	%RSE (95% CI)	Population estimate (BS estimate)	%RSE (95% CI)	Population estimate (BS estimate)	%RSE (95% CI)
Baseline hazard (day^−1^)	0.00600 (0.0103)	110 (0.00231–0.0406)	0.00120 0.00120	23.4 (0.000775–0.00199)	0.00168 0.00264	158 (0.000370–0.00893)	0.00324 0.00329	30.8 (0.00149–0.00535)
Hazard half-life (day)	12.8 (14.3)	67.4 (6.92–31.8)	—		—		—	
IC_50_ (ng/ml)	92.6 (94.7)	56.0 (10.3–202)	194 (465)	457 (35.2–2,410)	9.79 (18.5)	162 (0.703–87.1)	1,580 (863)	38.4 (307–1,600)
Slope	1.87 (2.98)	181 (0.979–9.46)	—		—		—	

The relative standard error (%RSE) was calculated as $100\times \frac{Standard\;deviation}{Average\;parameter\;estimate}$ from 1,000 iterations of a non-parametric bootstrap (BS) procedure.

IC_50_, half maximal inhibitory concentration.

To avoid these potential biases, 3 separate pharmacokinetic-pharmacodynamic time-to-event models were used to evaluate 1,210 African children weighing <15 kg (*n =* 36; 2.98% recrudescence), 638 African children weighing 15–25 kg (*n =* 7; 1.10% recrudescence), and 113 Southeast Asian pregnant women (*n =* 11; 9.73% recrudescence) (Fig 4; Table 3). Data from Southeast Asian pregnant women and African children were best described using a constant baseline hazard model with an *E*_MAX_ lumefantrine drug effect. All pharmacokinetic-pharmacodynamic time-to-event models in these particular populations displayed accurate predictive power (Fig 4) in the visual predictive check, although parameter estimates lacked precision and accuracy (Table 3). Moreover, pregnancy was not a statistically significant covariate when data from pregnant and (matched) non-pregnant women in Southeast Asia only were analysed. Furthermore, no statistically significant covariates were found in the pharmacokinetic-pharmacodynamic time-to-event model in African children.

### In silico dose optimization

**Fig 5:**
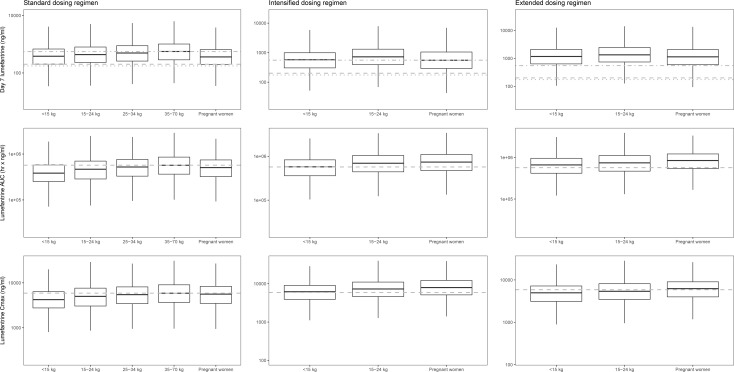
In silico dose optimisations using Monte Carlo simulations (*n =* 2,000) with the final lumefantrine pharmacokinetic model for the different populations consisting of children weighing <15 kg, 15–24 kg, and 25–34 kg; non-pregnant adults ≥35 kg; and pregnant women. The left, middle, and right column represent the results after a standard, intensified, and extended dosing regimen, respectively. The boxes and whiskers represent 25%–75% and 2.5%–97.5% of the data, respectively. The horizontal dashed-dotted grey line in the upper panels represents the median lumefantrine concentration at day 7 after standard treatment in non-pregnant adult patients (801 ng/ml). The dashed and dotted grey horizontal lines in the upper panels represent previously defined lumefantrine day 7 target concentrations of 175 and 200 ng/ml [16,23]. The horizontal grey dashed lines in the middle and lower panels represent the median lumefantrine area under the curve (AUC) (647,025 h × ng/ml) and maximum concentration (*C*_MAX_) (6,731 ng/ml) after standard treatment in a non-pregnant adult patient population.

As lumefantrine exposures were lower in young children and pregnant women, 3 alternative dosing regimens were evaluated and compared. A dose increase for pregnant women in their second and third trimester (100 mg artemether and 600 mg lumefantrine twice daily for 3 days, i.e., 1 extra tablet per dose) and for children weighing between 5 kg and 25 kg (lumefantrine doses: 120 mg for children 5–6 kg, 180 mg for children 7–8 kg, 240 mg for children 9–13 kg, and 360 mg for children 14–23 kg, twice daily for 3 days) did not result in equivalent lumefantrine concentrations at day 7 compared to a non-pregnant adult population receiving the standard dose (S3 Fig). However, the intensified dosing regimen (standard dose at 0, 8, 16, 24, 32, 40, 48, 56, and 64 hours) resulted in similar lumefantrine concentrations at day 7 compared to a non-pregnant adult population receiving the standard treatment (Fig 5). An extended dosing regimen (standard dose twice daily for a total 5 days) displayed the highest probability of target attainment, with >75% of the simulated lumefantrine concentrations at day 7 above the mean lumefantrine concentration at day 7 in a non-pregnant adult population receiving standard treatment (Fig 5). Total exposure and maximum lumefantrine concentrations were similar after intensified (thrice daily) and extended (5 day) dosing regimens and were substantially higher than those with an increased dosing regimen (Figs 5 and S3).

### Lumefantrine/desbutyl-lumefantrine pharmacokinetic model

The lumefantrine/desbutyl-lumefantrine pharmacokinetic model was developed based on data from 159 patients contributing 2 or more venous plasma samples per patient with a wide range of covariates (Table 1). A 2-compartment distribution model provided a substantial improvement of the model fit (*p <* 0.001; Δ−2LL = −281; ΔAIC = −277) compared to a 1-compartment distribution model, but the addition of a third disposition compartment did not improve the model further (*p >* 0.01; Δ−2LL = −8.68; ΔAIC = −4.68) (Table 4). Apart from the effect of body weight on clearance and volume parameters, no further covariates were included in the model. Unfortunately, a robust evaluation of pregnancy was not possible due to the small sample size (Table 1). The basic goodness-of-fit plots and parameter estimates showed a robust model without indications of model misspecification (S4 Fig). The predictive power was accurate considering the sampling design and sample size (Figs 2 and S5).

**Table 4:** Population parameter estimates from the simultaneous pharmacokinetic lumefantrine/desbutyl-lumefantrine drug-metabolite model.

Parameter	Fixed effects		Random effects	
	Population estimate (BS estimate)	%RSE (95% CI parameter estimate)	%CV for IIV (BS estimate)	%RSE (95% CI parameter estimate)
*F*	1 (fixed)	—	57.9 (57.2)	57.2 (49.0 to 64.9)
Box–Cox shape parameter on *F*	−0.449 (−0.459)	35.5 (−0.793 to −0.126)	—	—
*k*_a_ (h^−1^)	0.0409 (0.0422)	13.3 (0.0349 to 0.0567)	—	—
CL_LF_/*F* (l/h)	1.56 (1.57)	6.24 (1.40 to 1.77)	—	—
*V*_C LF_/*F* (l)	21.2 (21.6)	19.5 (14.6 to 30.5)	111 (110)	110 (87.5 to 134)
*Q*_LF_/*F* (l/h)	0.381 (0.387)	11.3 (0.313 to 0.497)	—	—
*V*_P LF_/*F* (l)	53.8 (54.5)	8.60 (46.2 to 65.2)	—	—
CL_DLF_/*F* (l/h)	78.4 (77.4)	7.80 (64.6 to 88.5)	38.8 (39.8)	39.8 (32.0 to 51.0)
*V*_C DLF_/*F* (l)	2,470 (2,560)	13.1 (1,960 to 3,280)	86.0 (82.3)	82.3 (49.5 to 110)
*Q*_DLF_/*F* (l/h)	104 (103)	13.2 (78.3 to 131)	34.9 (33.8)	33.8 (0.339 to 53.0)
*V*_P DLF_/*F* (l)	8,650 (8,870)	14.2 (6,990 to 11,800)	47.7 (45.3)	45.3 (0.453 to 68.9)
Dose_50_ (mg/kg) on *F*	3.86 (fixed)	—	—	—
Pregnancy on *k*_a_	0.513 (0.543)	52.4 (0.264 to 0.889)	—	—
Parasitaemia on *F*	−0.226 (−0.218)	81.8 (−0.557 to 0.156)	—	—
σ_LF_	0.251 (0.251)	11.8 (0.196 to 0.315)	—	—
σ_DLF_	0.0560 (0.0559)	8.46 (0.0467 to 0.0657)	—	—

Coefficient of variation (%CV) for inter-individual variability (IIV) was calculated as $100\times \sqrt{e^{estimate}-1}$. The relative standard error (%RSE) was calculated as $100\times \frac{Standard\;deviation}{Average\;parameter\;estimate}$ from 1,000 iterations of a non-parametric bootstrap (BS) procedure. The 95% confidence interval (95% CI) is displayed as the 2.5th to 97.5th percentile of the BS estimate, and the BS estimate as the average value.

LF: lumefantrine; DLF: desbutyl-lumefantrine; *F*: relative bioavailability; Box–Cox shape parameter: shape parameter on Box–Cox transformation; *k*_a_: absorption rate constant; *V*_C_/*F*: apparent central volume of distribution; *V*_P_/*F*: apparent peripheral volume of distribution; *Q*/*F*: inter-compartmental clearance; CL/*F*: elimination clearance; and σ: residual error additive on log scale. All parameters were centred around a non-pregnant patient with an admission parasitaemia of 15,800 parasites/μl, and clearance and volume parameters were centred around a non-pregnant patient weighing 42 kg and scaled allometrically ($CL/Q\;=\;\theta (n)\times {\frac{WT}{42}}^{\frac{3}{4}},\;V=\theta (n)\times \frac{WT}{42}$); parasitaemia was coded as in its logarithm in an exponential relationship centred around the mean ($F=\theta (n)\times e^{\theta_{parasitaemia}-(parasitaemia-4.2)}$), and a categorical pregnancy effect on *k*_a_ was coded as follows: *k*_a_ = θ × (1 + θ_pregnant_).

Pregnancy was a categorical/proportional covariate on *F* [1 + θ], dose_50_ was implemented using a saturation model on *F* [1 − (Dose/(θ + Dose))], and admission parasitaemia was implemented using a power relationship on *F* [(parasitaemia/median value)^θ^].

More technical information regarding the lumefantrine/desbutyl-lumefantrine pharmacokinetic model can be found in S2 Text.

## Discussion

The population pharmacokinetic model based on this covariate-rich dataset from nearly 4,000 patients provides an improved understanding of how body weight, pregnancy, dosage, and admission parasitaemia affect the absorption, distribution, and elimination of the most important and widely used anti-malarial therapy in current use. By applying a pharmacokinetic-pharmacodynamic time-to-event model to this dataset, we have made, to the best of our knowledge, the first comprehensive attempt to evaluate the relationship between pharmacokinetic factors and AL treatment outcome (PCR-corrected recrudescent malaria infections) across different geographical areas and populations. The lumefantrine/desbutyl-lumefantrine population pharmacokinetic model provides an improved understanding of the disposition effect of lumefantrine’s main active metabolite, which had hitherto remained poorly characterised. Young children and pregnant women have lower lumefantrine exposures compared with non-pregnant adults. The reason that young children have relatively low lumefantrine exposure is that currently recommended AL dosage regimens do not adjust adequately for the non-linear relationship between body weight and systemic exposure. Pregnant women were underexposed due to changes in the distribution kinetics of lumefantrine. Underexposure in these vulnerable populations contributes to lower cure rates and the selection of parasite resistance. In silico dose optimisations utilising the lumefantrine population pharmacokinetic model provide a sound basis for proposing improved dosing regimens for these 2 vulnerable groups.

### Lumefantrine pharmacokinetic model

Lumefantrine exposure and day 7 concentrations decreased substantially with increasing baseline parasite densities [16]. Higher parasitaemias reflect more severe disease, which could reduce the absorption as a result of reductions in visceral blood flow. The dose dependency of lumefantrine absorption was consistent with a previous study from the Thailand–Myanmar border in which lumefantrine exposure in patients with uncomplicated malaria was 30% lower when once-daily doses were administered compared to when the same dose was divided between 2 daily doses [14].

Weight-for-age *z-*score did not significantly improve the model fit when embedded in absorption, clearance, or distribution parameters. However, a pooled analysis on lumefantrine treatment outcome and day 7 concentration data showed a substantially higher risk of recrudescent malaria and lower concentrations at day 7 with decreasing weight-for-age *z-*scores [16,46]. Most probably the discrepancy between these 2 findings could be explained by the distribution of weight-for-age *z-*scores, which in this study had a large proportion of well-nourished (*z-*score between −2 and +2) children (214/281 children <5 years and 108/139 children <3 years). Moreover, weight-for-age *z-*score is an anthropometric indicator of both long-term (stunting) and short-term (wasting) nutritional status. The majority of the population studied here did not suffer from malnutrition, which could have affected significantly absorption, in contrast to the larger sample size studied previously. Weight-for-height *z-*scores may be a superior proxy for acute global malnutrition, especially in relation to drug pharmacology, but height was not available for all patients in the database.

Including age as a maturation factor for lumefantrine elimination clearance did not significantly improve the model fit. Maturation of enzymatic activity occurs during the first 2 years [12], and the lack of data in the youngest children (in the model building dataset the youngest child was 6 months old and only 4 patients were below 1 year) might explain the absence of this covariate relationship.

An earlier meta-analysis of observed lumefantrine concentrations at day 7 that included 2,787 patients indicated substantially lower lumefantrine concentrations on day 7 in patients with documented fever (i.e., >37.5°C admission axillary temperature) compared to patients without fever [16]. Also, filter paper dried capillary blood lumefantrine concentrations at day 7 were lower with increasing haemoglobin levels in the same analysis [16]. Admission body temperature and haemoglobin were evaluated here in a separate covariate analysis on a subset of data, but were not included in the final lumefantrine population pharmacokinetic model since these covariates were missing for more than 20% of the patients. In the patients in whom it was recorded, admission body temperature did not correlate significantly with any lumefantrine pharmacokinetic parameter in the current population pharmacokinetic model. The haemoglobin concentration did correlate with lumefantrine inter-compartmental clearance and apparent peripheral distribution volume, and, in contrast to previously published work, the results suggested somewhat higher lumefantrine concentrations at day 7 with increasing haemoglobin levels. However, the sampling matrix in this study was venous plasma as opposed to whole blood in filter paper, as in the previously reported study. Results should therefore be interpreted with caution.

Potential differences in lumefantrine distribution in venous blood and capillary blood when compared to venous plasma might not have been captured as venous and capillary blood samples were taken only at 7 days after treatment initiation. Thus, the reported proportional differences should be interpreted with caution. Increased lumefantrine exposures with crushed and dispersible tablets were in line with published results in healthy volunteers [47]. However, similarly to the interpretation of matrix effects, proportional differences for formulation effects should be interpreted cautiously as the sparse sampling design (i.e., 1 sample per patient) prevented formal evaluation of bioequivalence. Matrix and formulation effects need further evaluation in prospective clinical studies before firm conclusions can be drawn.

### Lumefantrine pharmacokinetic-pharmacodynamic time-to-event model

No statistically significant clinical covariates could be found in the pharmacokinetic-pharmacodynamic time-to-event model. This indicated that treatment failures in vulnerable patient groups (e.g., children below 5 years of age and pregnant women) could be explained fully by reduced lumefantrine exposures.

However, the artemether/dihydroartemisinin drug effect was not included in the lumefantrine pharmacokinetic-pharmacodynamic time-to-event model nor was a desbutyl-lumefantrine effect [48]. Consequently, all pharmacodynamic parameters, including baseline hazard and hazard half-life, are apparent, as the lumefantrine drug effect in the current pharmacokinetic-pharmacodynamic time-to-event model actually represents the sum of the artemether/dihydroartemisinin and lumefantrine/desbutyl-lumefantrine drug effects. Thus, the developed time-to-event model was not used for in silico dose optimisations because of the above issue in combination with poor parameter precision and accuracy of the pharmacokinetic-pharmacodynamic lumefantrine model.

### In silico dose optimisation

The high cure rates and excellent tolerability observed in non-pregnant adult patient populations suggest that conventional dosing in this group results in drug exposures within acceptably safe and effective therapeutic margins in most patients. Therefore, it is reasonable that dosage recommendations for other groups (including children and pregnant women) should be based on therapeutic targets that aim to achieve similar lumefantrine exposure (AUC and day 7 concentrations) to that of non-pregnant adults. This could improve cure rates in these groups and reduce the risks of drug resistance that could shorten AL’s useful therapeutic life [2].

Administering higher individual doses at the currently recommended frequency and duration (i.e., by adding extra tablets to each dose in the usual twice-daily regimen) failed to result in proportional linear increases in overall drug exposures across all target populations. This presumably reflects dose-limited absorption. We modelled an additional 2 alternative dosing regimens: an intensified regimen (thrice-daily dosing with the currently recommended weight-based number of tablets per dose) and an extended regimen (with the currently recommended weight-based number of tablets per dose given twice daily for 5 days). Enrolment parasite density was not taken into account in dose optimisation simulations since quantification of parasites at enrolment is not always possible. Both alternative dosing regimens resulted in similar or higher lumefantrine concentrations on day 7 and AUCs than in non-pregnant adults given the currently recommended standard dosing regimen. However, higher maximum concentrations were not seen in these simulations, suggesting that pregnant women and young children receiving these regimens would not face added risks of peak drug exposure-related acute toxicity, although further investigation in prospective clinical studies would be required to confirm this [6].

From a pharmacological perspective, a dose extension (i.e., twice-daily dosing at the current dosage for 5 days) for children ≤25 kg and pregnant women during the second and third trimester probably has the greatest advantages for therapeutic efficacy. A dose extension would result not only in adequate lumefantrine exposures but also, even more importantly, in an additional malaria asexual replication cycle being exposed to artemether/dihydroartemisinin. This would contribute to a lower parasite biomass. Unfortunately, artemether/dihydroartemisinin concentration-time data were not available in this study, which prevented us from formal clinical trial simulations using a pharmacokinetic-pharmacodynamic time-to-event approach for PCR-confirmed recrudescent malaria at day 42.

An extended dosing regimen needs to be considered in light of greater challenges in ensuring adherence to longer courses of anti-malarials. A 4-day treatment might be an option if a 5-day treatment is not possible due to poor adherence and/or cost issues. The alternative approach of intensifying frequency may also have programmatic disadvantages including dosing compliance with a more complex regimen and the need for repackaging, which increases the burden on pharmaceutical companies. The current development of a new formulation that provides increased lumefantrine absorption in animal and healthy volunteer studies might provide a solution [49,50]. Nevertheless, until this formulation is commercially available, the efficacy, safety, and tolerability of the proposed regimens with the conventional formulation in children and pregnant women will require further evaluation in prospective clinical trials. Moreover, the population pharmacokinetic model developed in this study can be used for dose optimisation of the novel formulation as lumefantrine metabolism and elimination will remain the same.

### Study limitations

The developed lumefantrine population pharmacokinetic model and subsequent evaluation of alternative dosing regimens have a number of limitations. Model development was conducted using venous plasma data from patients receiving intact tablets administered with fat. Simulations, including evaluations and comparisons of alternative dosing regimens, are consequently representative only for venous plasma after administration of intact tablets with fat. Adjustments for sampling matrix (i.e., venous blood, capillary blood, or capillary plasma) or formulation (i.e., crushed or dispersible tablets) were not performed since correction factors were not considered reliable due to the sparseness of data. Furthermore, all patients included in this analysis received AL with fat, eliminating the possibility to characterise and quantify the impact of concomitant fat intake on the bioavailability of lumefantrine. The absence of artemether-dihydroartemisinin data prevented a full evaluation of AL therapy. Consequently, lumefantrine concentrations at day 7 were used as a pharmacokinetic endpoint. Nevertheless, the model remains clinically relevant considering that the vast majority of patients are treated with intact tablets co-administered with fat.

After the database was closed, 7 studies meeting the inclusion criteria were published (S2 Table) [10,12,13,51–54]. Two studies in Malawi and Uganda evaluated lumefantrine pharmacokinetic drug–drug interactions with anti-retroviral therapies for HIV-co-infected paediatric patients [52,54]. Drug–drug interaction data were not available in the pooled lumefantrine pharmacokinetic database, and therefore, these data would not have made a substantial impact on the analysis presented here. A total of 3 studies in pregnant women with uncomplicated *P*. *falciparum* malaria in Tanzania and Uganda were identified [10,13,53]. All women studied were in their second and third trimester. Pregnant women in Tanzania and Uganda displayed a similar pattern of decreased lumefantrine exposure compared to non-pregnant women as in this pooled analysis [10,53]. Unlike this pooled analysis, a study in Uganda did not report significant differences in lumefantrine exposure in pregnant and non-pregnant women, but a less powerful non-parametric, non-compartmental analysis was used, which could potentially explain this discrepancy [13]. A study in Mali and Niger in severely malnourished children (weight-for-height *z-*score < –3) between 6 months and 5 years found lower lumefantrine concentrations at day 7 compared to matched nourished children (weight-for-height *z-*score ≥ –3) [51]. This pattern of lumefantrine underexposure with malnourishment was not apparent in this pooled study, a finding most likely explained by a discrepancy in *z-*score distributions, with a larger proportion of well-nourished (*z-*score between −2 and +2) children (214/281 children <5 years and 108/139 children <3 years) in the pooled analysis presented here. The impact of malnutrition on lumefantrine exposure needs to be evaluated further in a large pooled analysis including severely, moderately, and non-malnourished children from different regions. In line with the results presented here, a paediatric study in Uganda reported body weight as an allometric covariate for clearance and volume parameters, resulting in relatively lower lumefantrine exposure in small children [12]. However, a positive correlation between lumefantrine exposure and age was also identified in the children in Uganda. In the pooled analysis presented here, baseline parasitaemia displayed a negative correlation with exposure. Thus, these correlated covariates (age and baseline parasitaemia correlate negatively) might be a likely explanation for the apparent discrepancy between the 2 studies.

### Conclusions

Conventional weight-based dosing in young children (≤25 kg) and pregnant women resulted in significantly lower lumefantrine drug exposures when compared to non-pregnant adults, and this may underpin poorer cure rates in these particularly vulnerable groups. Substantial dose-limited absorption of lumefantrine using the currently available formulation limits opportunities to improve drug exposure in these groups by just escalating the amount of drug administered with each dose. However, pharmacokinetic model-based simulations of alternative strategies suggest that, assuming similar adherence, intensified (3 times daily for 3 days) or extended (2 times daily for 5 days) dose regimens could result in equivalent lumefantrine exposures to those of non-pregnant adults treated conventionally. An extended dosing regimen is most favourable from a pharmacological perspective as an additional malaria asexual replication cycle is thereby exposed to artemether/dihydroartemisinin, which will undoubtedly contribute to increased parasitological killing. Given that AL is now the most widely used drug for treating the global burden of malaria illness, estimated at >200 million cases annually, prospective clinical dose optimisation studies evaluating efficacy, safety, and tolerability, as well as both artemether/dihydroartemisinin and lumefantrine/desbutyl-lumefantrine pharmacokinetics, are now warranted in order to provide in vivo confirmation of our in silico findings for these proposed dosing regimens. Future research should also include evaluations of the regimens’ acceptability and adherence in order to determine the feasibility, practicality, and effectiveness of longer or more intense regimens. Implementing improved dosing regimens may have implications for reducing treatment failures in these vulnerable populations. Young African children represent the largest and most important population affected by malaria, and pregnant women are at higher risk of severe complications, death, and adverse pregnancy outcomes due to malaria. Improved dosing also has the potential benefit of limiting sub-therapeutic drug exposures at a population level, thereby reducing the risk of drug resistance and extending the therapeutic life of this important artemisinin-based combination treatment.

## Supporting information

S1 PRISMA Checklist (DOCX)

S1 Fig Goodness-of-fit diagnostics for lumefantrine when fitting the final lumefantrine pharmacokinetic model. The black solid line, black dashed line, and open circles represent the line of identity, trend line, and observations, respectively.
(TIF)

S2 Fig External validation and alternative sampling matrix prediction-corrected visual predictive checks. External validation using prediction-corrected visual predictive check of sparse venous plasma sampling after intact tablets (A). Prediction-corrected visual predictive check of sparse venous plasma sampling data after dispersible tablets (B), sparse venous plasma sampling data after crushed tablets (C), sparse venous blood sampling data (D), sparse capillary blood sampling data (E), and dense capillary plasma sampling data (F). Open circles represent observed lumefantrine concentrations. Solid lines represent the 5th, 50th, and 95th percentiles of the observed data. Grey shaded areas represent the 95% confidence intervals of the 5th, 50th, and 95th percentiles of the simulated (*n =* 2,000) data.
(TIF)

S3 Fig In silico dose optimisations using Monte Carlo simulations (*n =* 2,000) with the lumefantrine model. The boxes and whiskers represent 25%–75% and 2.5%–97.5% of the data, respectively. The horizontal dashed-dotted grey line in the upper panel represents the median lumefantrine concentration at day 7 after standard treatment in a non-pregnant adult patient population (801 ng/ml). The dashed and dotted grey horizontal lines in the upper panel represent previously defined lumefantrine day 7 target concentrations of 175 and 200 ng/ml [4,9]. The horizontal grey dashed lines in the middle and lower panels represent the median lumefantrine area under the curve (AUC) (647,025 h × ng/ml) and maximum concentration (*C*_MAX_) (6,731 ng/ml) after standard treatment in a non-pregnant adult patient population.
(EPS)

S4 Fig Goodness-of-fit diagnostics for lumefantrine and desbutyl-lumefantrine when fitting the final pharmacokinetic drug-metabolite model. Lumefantrine (top); desbutyl-lumefantrine (bottom). The black solid line, black dashed line, and open circles represent the line of identity, trend line, and observations, respectively.
(TIF)

S5 Fig External validation using prediction-corrected visual predictive check of the sparse venous plasma sampling. Open circles represent the observed sparsely sampled desbutyl-lumefantrine venous plasma concentration data. Solid lines represent the 5th, 50th, and 95th percentiles of the observed data. Grey shaded areas represent the 95% confidence intervals of the 5th, 50th, and 95th percentiles of the simulated (*n =* 2,000) data.
(TIF)

S1 Table Demographic summary of studies included in the pooled analyses. (DOCX)

S2 Table Demographic summary of post-2013 studies meeting the original inclusion criteria. (DOCX)

S1 Text Supplementary methods: Detailed technical information regarding the pharmacokinetic model building process, the pharmacokinetic-pharmacodynamic time-to-event model building process, and dose optimisations. (DOCX)

S2 Text Supplementary results: Detailed technical information on the pharmacokinetic model and dose optimisation results. (DOCX)

## Abbreviations

ACT: artemisinin-based combination therapy
AL: artemether-lumefantrine
AUC: area under the curve
WWARN: WorldWide Antimalarial Resistance Network
