# Supplementary figures and images for: Artemether-lumefantrine dosing for malaria treatment in young children and pregnant women: A pharmacokinetic-pharmacodynamic meta-analysis
Source: PLoS Med. 2018 Jun 12;15(6):e1002579. doi: 10.1371/journal.pmed.1002579 (PMC5997317; doi:10.1371/journal.pmed.1002579)

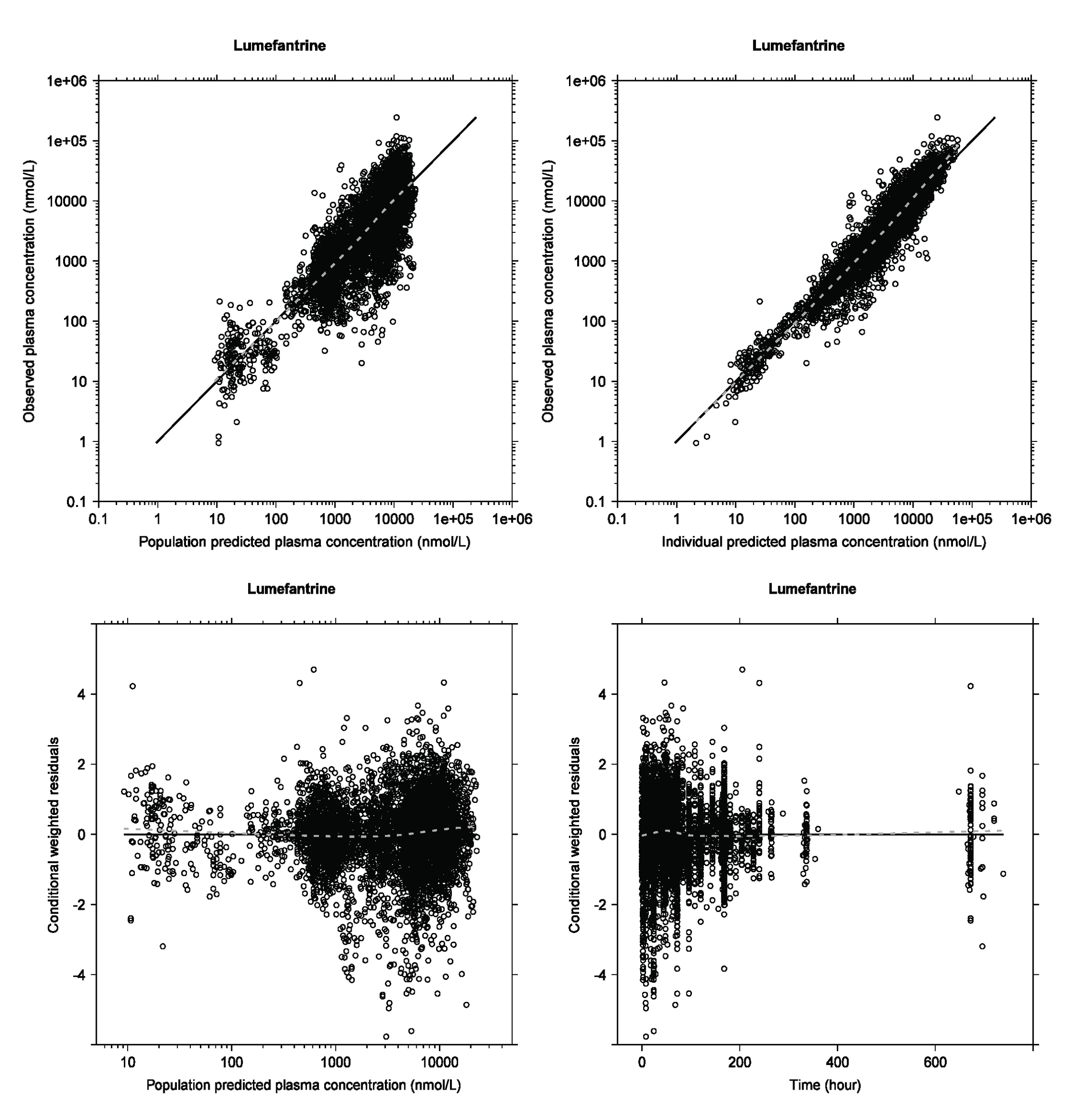

Supplement: S1 Fig — The black solid line, black dashed line, and open circles represent the line of identity, trend line, and observations, respectively. (TIF) [file pmed.1002579.s002.tif]

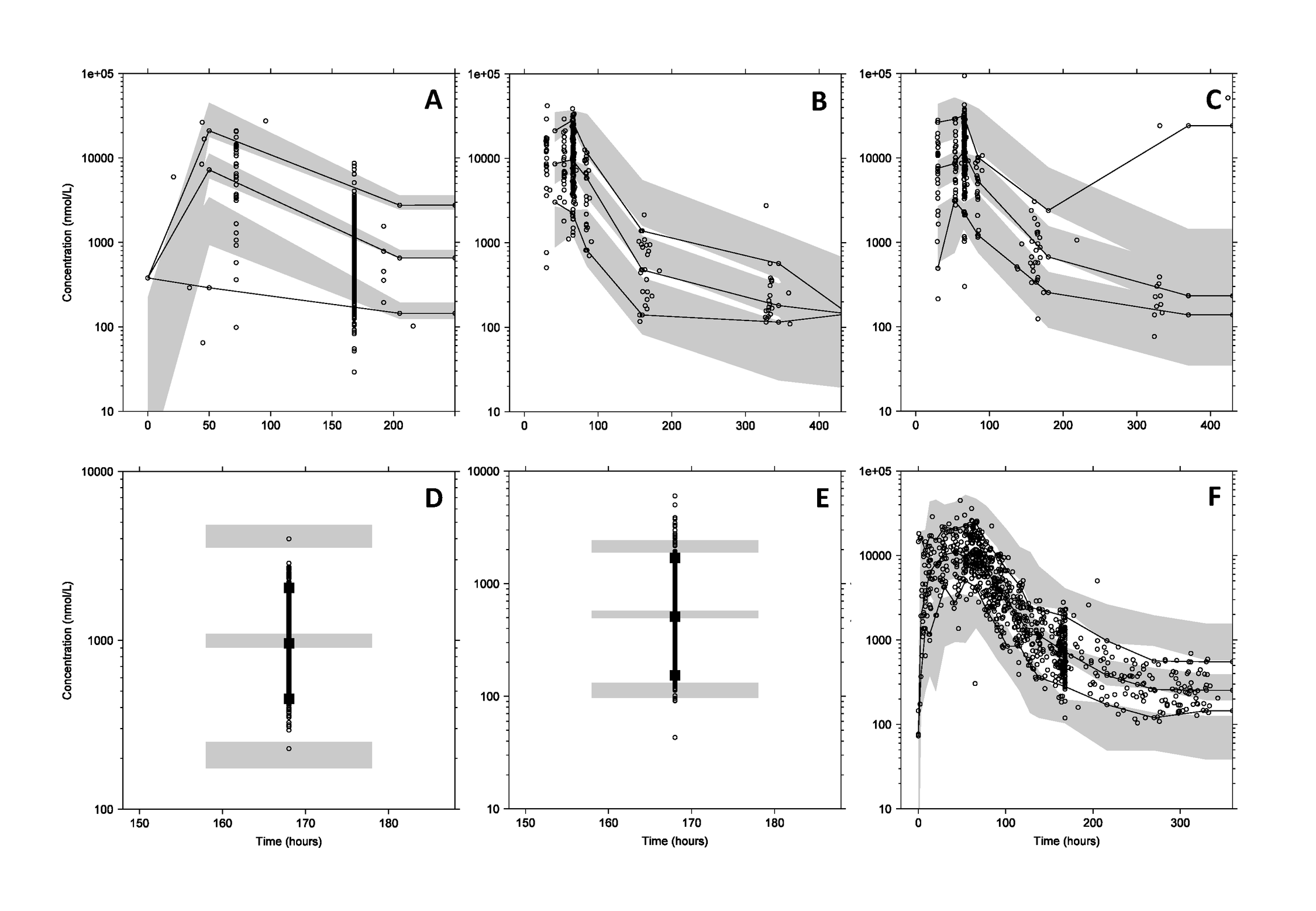

Supplement: S2 Fig — External validation using prediction-corrected visual predictive check of sparse venous plasma sampling after intact tablets (A). Prediction-corrected visual predictive check of sparse venous plasma sampling data after dispersible tablets (B), sparse venous plasma sampling data after crushed tablets (C), sparse venous blood sampling data (D), sparse capillary blood sampling data (E), and dense capillary plasma sampling data (F). Open circles represent observed lumefantrine concentrations. Solid lines represent the 5th, 50th, and 95th percentiles of the observed data. Grey shaded areas represent the 95% confidence intervals of the 5th, 50th, and 95th percentiles of the simulated (n = 2,000) data. (TIF) [file pmed.1002579.s003.tif]

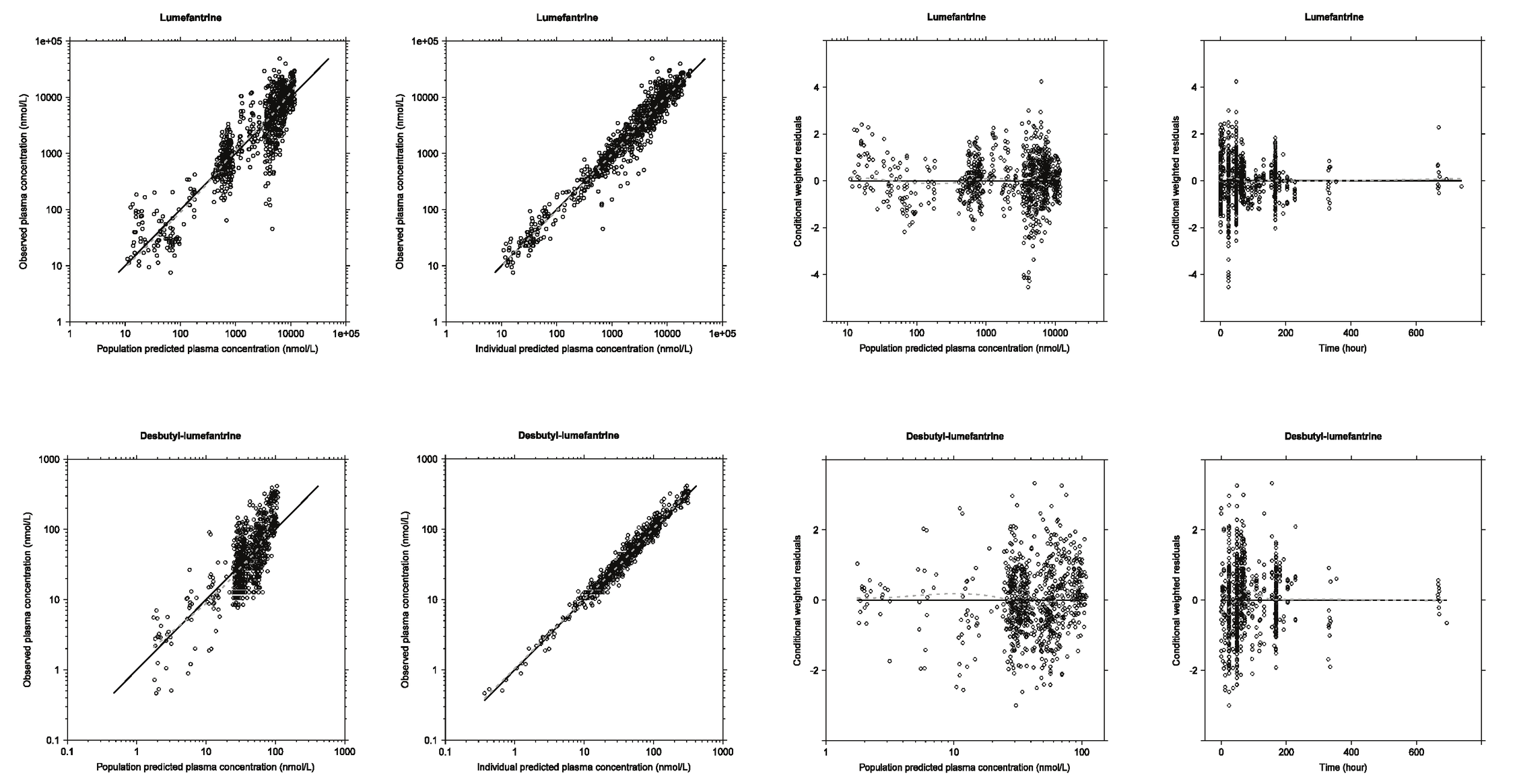

Supplement: S4 Fig — Lumefantrine (top); desbutyl-lumefantrine (bottom). The black solid line, black dashed line, and open circles represent the line of identity, trend line, and observations, respectively. (TIF) [file pmed.1002579.s005.tif]

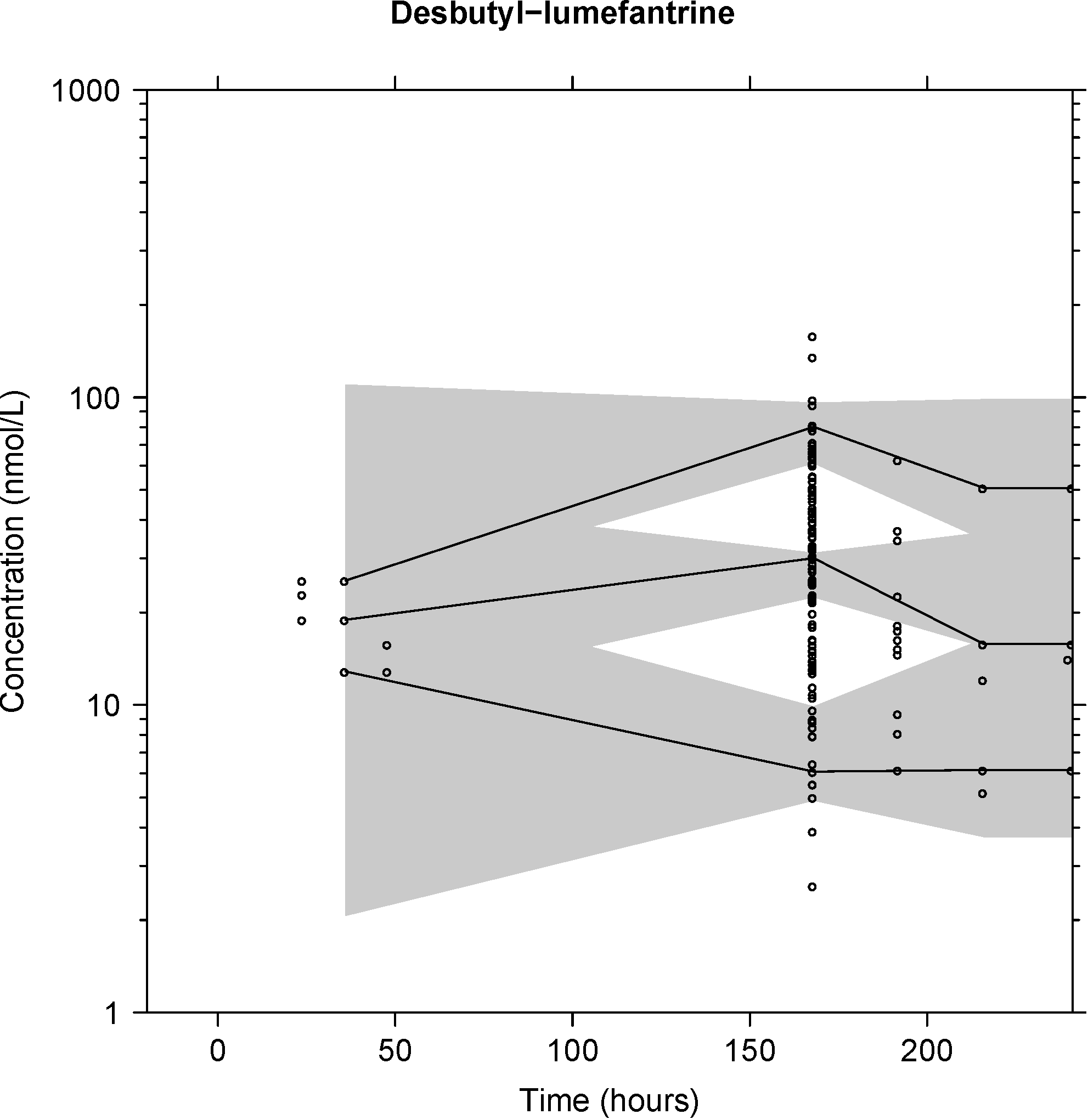

Supplement: S5 Fig — Open circles represent the observed sparsely sampled desbutyl-lumefantrine venous plasma concentration data. Solid lines represent the 5th, 50th, and 95th percentiles of the observed data. Grey shaded areas represent the 95% confidence intervals of the 5th, 50th, and 95th percentiles of the simulated (n = 2,000) data. (TIF) [file pmed.1002579.s006.tif]
