# Supplementary material for: Artemether-lumefantrine dosing for malaria treatment in young children and pregnant women: A pharmacokinetic-pharmacodynamic meta-analysis
Source: PLoS Med. 2018 Jun 12;15(6):e1002579. doi: 10.1371/journal.pmed.1002579 (PMC5997317; doi:10.1371/journal.pmed.1002579)
Supplement: S1 Table — (DOCX) [file pmed.1002579.s007.docx]

| **Table 1:** demographic summary of studies included in the pooled analyses. | | | | | | | | | | | | | | | | |
| --- | --- | --- | --- | --- | --- | --- | --- | --- | --- | --- | --- | --- | --- | --- | --- | --- |
| Reference | Study site | Study size | Dose regimens | Dose (mg/kg) | Sampling matrices | Sample size LF | Samples/patient LF | Sample size DLF | Samples/patient DLF | Male/female (%) | Age (years) | Bodyweight (kg) | Z-score (weight for age) | Parasitaemia | Pregnant women (%) | Estimated gestational age (weeks) |
| [[1](#_ENREF_1)] | 1, 5, 11, 13, 21 | 624 | 6 doses over 3 days | 12 [8.16-24: 10-14.1] | Venous plasma | 594 | 1 [1-1: 1-1] | - | - | 51.6/48.4 | 3.42 [0-12.4: 2.06-5.42] | 13 [5-34: 10.8-16.8] | -0.895 [-4.28-10.5: -1.64--0.215] | 28900 [520-629000: 10900-69200] | 0 | - |
| [[2](#_ENREF_2)] | 2 | 122 | 6 doses over 3 days | 12.3 [8.11-16.1: 10.9-13.6] | Capillary blood | 121 | 1 [1-1: 1-1] | - | - | 59/41 | 7.29 [0.361-14.6: 5-11] | 20 [7.5-50.5: 15.5-29] | -0.715 [-3.49-4.54: -1.41--0.18] | 25600 [1200-4e+05: 7990-67500] | 0 | - |
| [[3](#_ENREF_3)] | 3, 22 | 353 | 6 doses over 3 days | 12 [8.11-24: 10-13.3] | Capillary blood | 339 | 1 [1-1: 1-1] | - | - | 48.2/51.8 | 2.67 [0.333-5: 1.83-3.58] | 12 [5-21: 10-14] | -0.81 [-4.47-3.31: -1.51--0.02] | 48700 [2220-192000: 23300-86800] | 0 | - |
| [[3](#_ENREF_3)] | 3, 22 | 152 | 6 doses over 3 days | 12 [8.14-20: 10-13.3] | Capillary blood | 133 | 1 [1-1: 1-1] | - | - | 46.7/53.3 | 3 [0.417-5: 1.92-3.75] | 12 [6-21: 10-14.8] | -0.885 [-4.22-2: -1.54--0.0975] | 17400 [140-205000: 4840-49000] | 0 | - |
| [[4](#_ENREF_4)] | 3 | 50 | 6 doses over 3 days | 12 [8.57-16: 10.9-13.9] | Venous plasma | 404 | 8 [5-9: 8-9] | - | - | 38/62 | 4 [1-9.92: 2-6] | 12.6 [8-30: 10-16.8] | -1.28 [-3.75-1.72: -1.88--0.655] | 34800 [2120-192000: 5570-57500] | 0 | - |
| [[5](#_ENREF_5)] | 4 | 20 | 6 doses over 3 days | 11.6 [9.6-13.8: 11.2-12.2] | Venous plasma | 120 | 6 [6-6: 6-6] | - | - | 50/50 | 9.4 [5.07-12.4: 6.9-11.3] | 25.5 [20-41: 21-31] | -0.36 [-0.79-0.56: -0.572--0.27] | 19100 [480-147000: 3960-50600] | 0 | - |
| [[6](#_ENREF_6)] | 6 | 177 | 6 doses over 3 days | 12 [8.45-18.5: 10.9-15] | Capillary blood | 157 | 1 [1-1: 1-1] | - | - | 50.8/49.2 | 2.82 [0.497-5.17: 1.66-4.19] | 11 [6.5-19: 10-14] | -1.29 [-5.3-3.31: -2.38--0.46] | 31500 [480-2e+05: 4800-68800] | 0 | - |
| [[7](#_ENREF_7)] | 7 | 101 | 6 doses over 3 days | 10.6 [8.05-18.8: 9.16-13.4] | Venous plasma | 101 | 1 [1-1: 1-1] | - | - | 60.4/39.6 | 2.83 [0.5-4.92: 1.67-3.67] | 12 [6.4-18: 9.4-13.6] | -1.22 [-3.62-2.3: -1.87--0.52] | 57200 [2120-214000: 33800-113000] | 0 | - |
| [[8](#_ENREF_8)] | 8 | 143 | 6 doses over 3 days | 11.2 [3.2-18.5: 9.8-13.2] | Venous plasma | 486 | 3 [2-4: 3-4] | 392 | 3 [1-4: 3-3] | 43.4/56.6 | 9 [1-78: 3.75-15.5] | 20 [6.5-150: 13-41] | -1.59 [-4.1-1.08: -2.07--1.02] | 15400 [120-4e+05: 4220-57600] | 2.1 | - |
| [[9](#_ENREF_9)] | 9 | 125 | 6 doses over 3 days | 10 [5.26-12: 8.51-11.6] | Venous plasma | 212 | 2 [1-2: 1-2] | 121 | 1 [1-1: 1-1] | 55.2/44.8 | 2.92 [0.5-5: 1.92-4] | 11 [5.7-23: 9.5-13.5] | -1.52 [-5.31-2.62: -2.29--0.86] | 41400 [280-450000: 13300-101000] | 0 | - |
| [[10](#_ENREF_10)] | 10 | 13 | 6 doses over 3 days | 11.4 [8.89-12.6: 10.9-12] | Venous plasma | 201 | 16 [14-16: 15-16] | 197 | 16 [12-16: 15-16] | 61.5/38.5 | 7.58 [5.17-9.67: 6.75-9] | 19 [15-27: 16-21] | -1.73 [-3.43--0.65: -2.55--1.21] | 11400 [98-127000: 3190-64200] | 0 | - |
| [[11](#_ENREF_11)] | 12 | 470 | 6 doses over 3 days | 10.6 [5.39-16: 9.06-12] | Venous plasma | 887 | 2 [1-2: 2-2] | - | - | 47/53 | 9.01 [1.21-80: 4.31-20.1] | 24 [10-89: 15-49.4] | -0.84 [-4.39-2.56: -1.58--0.14] | 10500 [125-140000: 3460-32500] | 0.213 | - |
| [[12](#_ENREF_12)] | 12 | 116 | 6 doses over 3 days | 8.42 [5.78-10.9: 7.74-9.23] | Venous and capillary plasma | 1030 | 5 [3-26: 5-6] | - | - | 0/100 | 21.5 [15-38: 19-25] | 57 [44-83: 52-62] | - | 1380 [24-194000: 203-5140] | 100 | 22 [13-39: 19-30.2] |
| [[13](#_ENREF_13)] | 14 | 462 | 6 doses over 3 days | 10.9 [5.85-18.9: 9.6-12.4] | Venous blood | 440 | 1 [1-1: 1-1] | - | - | 49.1/50.9 | 13.1 [6.03-86.7: 9.79-18.7] | 34.5 [18-82: 24-50] | -1.24 [-3.14-5.73: -1.88--0.55] | 416 [16-285000: 112-1550] | 0 | - |
| [[14](#_ENREF_14)] | 14 | 141 | 6 doses over 3 days | 10.5 [8.05-17.1: 9.02-13.3] | Venous blood | 106 | 1 [1-1: 1-1] | - | - | 58.2/41.8 | 3.14 [0.745-5.01: 2.15-4.09] | 13 [7-21: 11.2-14.6] | -0.91 [-3.24-1.83: -1.59--0.07] | 26100 [2000-194000: 4800-48900] | 0 | - |
| [[15](#_ENREF_15)] | 15 | 77 | 6 doses over 3 days | 11.2 [6.67-16: 9.63-12.6] | Venous plasma | 77 | 1 [1-1: 1-1] | - | - | 50.6/49.4 | 12 [1-57: 5-25] | 28.5 [8-72: 15.5-47] | -1.15 [-4.97-3.45: -2.46--0.47] | 29000 [5280-202000: 10600-53400] | 0 | - |
| [[16](#_ENREF_16)] | 16, 18 | 44 | 6 doses over 3 days | 11.8 [8.05-16.7: 9.96-14.9] | Capillary blood | 34 | 1 [1-1: 1-1] | - | - | 43.2/56.8 | 1.75 [0.5-5: 1.13-3.37] | 11 [7.2-15.7: 8.98-12.7] | -0.91 [-3.11-1.89: -1.62--0.12] | 16000 [2080-524000: 5930-38200] | 0 | - |
| [[17](#_ENREF_17)] | 17 | 103 | 6 doses over 3 days | 9.8 [7.38-13.7: 9.06-10.3] | Capillary plasma | 466 | 5 [1-5: 4-5] | - | - | 0/100 | 24 [15-42: 20-33] | 49 [35-65: 46.5-53] | - | 3960 [56.8-154000: 668-21300] | 100 | 22.6 [13.1-39: 17.8-28.4] |
| [[18](#_ENREF_18)] | 17 | 225 | 6 doses over 3 or 5 days and 4 doses over 2 days | 9.6 [7.16-16: 9.06-10.9] | Venous plasma | 815 | 4 [1-5: 4-4] | - | - | 72/28 | 23 [3-75: 16-35] | 49 [10-67: 40-53] | -2.41 [-4.03--0.5: -2.77--1.78] | 5250 [381-2e+05: 1650-44000] | 0 | - |
| [[19](#_ENREF_19)] | 19 | 51 | 6 doses over 3 or 5 days and 4 doses over 2 days | 10.2 [6.96-13.3: 8.73-10.7] | Venous plasma | 633 | 13 [9-13: 12-13] | - | - | 64.7/35.3 | 23 [15-50: 20-30] | 47 [36-69: 44.8-55] | - | 13300 [529-176000: 4430-39200] | 0 | - |
| [[20](#_ENREF_20)] | 19, 17 | 147 | 6 doses over 3 days | 9.7 [5.93-16: 8.73-10.9] | Venous plasma | 715 | 5 [3-6: 5-5] | - | - | 72.8/27.2 | 22 [2-63: 16-30] | 49.5 [8-81: 41-55] | -1.34 [-3.07-2.08: -2.17--0.242] | 6260 [264-165000: 2410-34100] | 0 | - |
| [[21](#_ENREF_21)] | 20 | 79 | 6 doses over 3 days | 9.6 [7.27-14.1: 8.73-10.9] | Venous plasma | 79 | 1 [1-1: 1-1] | - | - | 81/19 | 23 [6-49: 18.5-29.5] | 50 [17-66: 41.5-55] | -2.28 [-3.12--1.45: -2.7--1.87] | 19600 [21.2-1e+05: 5930-43100] | 0 | - |
| [[22](#_ENREF_22)] | 17 | 36 | 6 doses over 3 days; 3 doses over 3 days | 15.5 [7.74-20.9: 9.6-17.5] | Venous plasma | 463 | 13 [4-14: 13-14] | - | - | 86.1/13.9 | 29.5 [18-64: 26.8-40] | 54 [42-63: 49.8-56.2] | - | 5120 [92.8-142000: 849-36400] | 0 | - |
| [[23](#_ENREF_23)] | 17 | 13 | 6 doses over 3 days | 10.2 [8.42-11.7: 9.8-10.7] | Venous plasma | 196 | 15 [15-16: 15-15] | 152 | 15 [10-15: 13.5-15] | 0/100 | 20 [14-42: 18-27] | 47 [41-57: 45-49] | - | 758 [91-25600: 299-2040] | 100 | 23 [13.1-38: 19.2-30.1] |
| [[24](#_ENREF_24)] | 17 | 44 | 4 doses over 2 days | 9.51 [7.5-11.7: 8.73-10.6] | Venous plasma | 44 | 1 [1-1: 1-1] | - | - | 84.1/15.9 | 25.5 [9-63: 17.8-37] | 49.5 [22-64: 42-55] | -1.33 [-1.74--0.92: -1.54--1.12] | 2390 [43-161000: 625-41900] | 0 | - |
| [[25](#_ENREF_25)] | 19 | 164 | 6 doses over 3 days | 9.52 [5.91-13.7: 8.72-10.4] | Venous plasma | 449 | 2 [1-6: 2-3] | - | - | 70.1/29.9 | 25 [12-71: 20.8-34] | 50.4 [35-81.2: 46-55] | - | 1610 [13-436000: 194-20500] | 0 | - |

*Data are presented as median [range: interquartile range].*

*Included study sites were Benin (1); Bandim Health Projects study area (Bandim, Belem, and Cuntum), Guinea-Bissau (2); Fukayosi, Tanzania (3); Kampala, Uganda (4); Kenya (5); Kibaha District, Tanzania (6); Kilifi, Kenya (7); Kilombero District, Tanzania (8); Madang and East Sepik Provinces, Papua New Guinea (9); Madang Province, Papua New Guinea (10); Mali (11); Mbarara, Uganda (12); Mozambique, Mozambique (13); Nimba County, Liberia (14); Phalanxay District, Loas (15); Sekou, Liberia (16); SMRU, Thailand (17); Allada, Benin (18); Bangkok, Thailand (19); Battambang Province, Cambodia (20); Tanzania (21) and Yombo, Tanzania (22). Age-for-weight z-score was calculated for all children below 10.08 years of age*.

## References

1. Djimde AA, Tekete M, Abdulla S, Lyimo J, Bassat Q, Mandomando I, et al. Pharmacokinetic and pharmacodynamic characteristics of a new pediatric formulation of artemether-lumefantrine in African children with uncomplicated Plasmodium falciparum malaria. Antimicrobial agents and chemotherapy. 2011;55(9):3994-9. doi: 10.1128/AAC.01115-10. PubMed PMID: 21670177; PubMed Central PMCID: PMC3165288.

2. Ursing J, Kofoed PE, Rodrigues A, Blessborn D, Thoft-Nielsen R, Bjorkman A, et al. Similar efficacy and tolerability of double-dose chloroquine and artemether-lumefantrine for treatment of Plasmodium falciparum infection in Guinea-Bissau: a randomized trial. The Journal of infectious diseases. 2011;203(1):109-16. doi: 10.1093/infdis/jiq001. PubMed PMID: 21148503; PubMed Central PMCID: PMC3086436.

3. Ngasala BE, Malmberg M, Carlsson AM, Ferreira PE, Petzold MG, Blessborn D, et al. Efficacy and effectiveness of artemether-lumefantrine after initial and repeated treatment in children <5 years of age with acute uncomplicated Plasmodium falciparum malaria in rural Tanzania: a randomized trial. Clin Infect Dis. 2011;52(7):873-82. doi: 10.1093/cid/cir066. PubMed PMID: 21427394.

4. Hietala SF, Martensson A, Ngasala B, Dahlstrom S, Lindegardh N, Annerberg A, et al. Population pharmacokinetics and pharmacodynamics of artemether and lumefantrine during combination treatment in children with uncomplicated falciparum malaria in Tanzania. Antimicrobial agents and chemotherapy. 2010;54(11):4780-8. doi: 10.1128/AAC.00252-10. PubMed PMID: 20713675; PubMed Central PMCID: PMC2976134.

5. Mwesigwa J, Parikh S, McGee B, German P, Drysdale T, Kalyango JN, et al. Pharmacokinetics of artemether-lumefantrine and artesunate-amodiaquine in children in Kampala, Uganda. Antimicrobial agents and chemotherapy. 2010;54(1):52-9. doi: 10.1128/AAC.00679-09. PubMed PMID: 19841149; PubMed Central PMCID: PMC2798532.

6. Ngasala BE, Malmberg M, Carlsson AM, Ferreira PE, Petzold MG, Blessborn D, et al. Effectiveness of artemether-lumefantrine provided by community health workers in under-five children with uncomplicated malaria in rural Tanzania: an open label prospective study. Malar J. 2011;10:64. doi: 10.1186/1475-2875-10-64. PubMed PMID: 21410954; PubMed Central PMCID: PMC3065443.

7. Borrmann S, Sasi P, Mwai L, Bashraheil M, Abdallah A, Muriithi S, et al. Declining responsiveness of Plasmodium falciparum infections to artemisinin-based combination treatments on the Kenyan coast. PloS one. 2011;6(11):e26005. doi: 10.1371/journal.pone.0026005. PubMed PMID: 22102856; PubMed Central PMCID: PMC3213089.

8. Hodel EM, Kabanywanyi AM, Malila A, Zanolari B, Mercier T, Beck HP, et al. Residual antimalarials in malaria patients from Tanzania--implications on drug efficacy assessment and spread of parasite resistance. PloS one. 2009;4(12):e8184. doi: 10.1371/journal.pone.0008184. PubMed PMID: 20011529; PubMed Central PMCID: PMC2788605.

9. Karunajeewa HA, Mueller I, Senn M, Lin E, Law I, Gomorrai PS, et al. A trial of combination antimalarial therapies in children from Papua New Guinea. The New England journal of medicine. 2008;359(24):2545-57. doi: 10.1056/NEJMoa0804915. PubMed PMID: 19064624.

10. Salman S, Page-Sharp M, Griffin S, Kose K, Siba PM, Ilett KF, et al. Population pharmacokinetics of artemether, lumefantrine, and their respective metabolites in Papua New Guinean children with uncomplicated malaria. Antimicrobial agents and chemotherapy. 2011;55(11):5306-13. Epub 2011/08/31. doi: 10.1128/AAC.05136-11. PubMed PMID: 21876056; PubMed Central PMCID: PMC3194999.

11. Piola P, Fogg C, Bajunirwe F, Biraro S, Grandesso F, Ruzagira E, et al. Supervised versus unsupervised intake of six-dose artemether-lumefantrine for treatment of acute, uncomplicated Plasmodium falciparum malaria in Mbarara, Uganda: a randomised trial. Lancet. 2005;365(9469):1467-73. doi: 10.1016/S0140-6736(05)66416-1. PubMed PMID: 15850630.

12. Piola P, Nabasumba C, Turyakira E, Dhorda M, Lindegardh N, Nyehangane D, et al. Efficacy and safety of artemether-lumefantrine compared with quinine in pregnant women with uncomplicated Plasmodium falciparum malaria: an open-label, randomised, non-inferiority trial. The Lancet Infectious diseases. 2010;10(11):762-9. doi: 10.1016/S1473-3099(10)70202-4. PubMed PMID: 20932805.

13. Schramm B, Valeh P, Baudin E, Mazinda CS, Smith R, Pinoges L, et al. Tolerability and safety of artesunate-amodiaquine and artemether-lumefantrine fixed dose combinations for the treatment of uncomplicated Plasmodium falciparum malaria: two open-label, randomized trials in Nimba County, Liberia. Malar J. 2013;12:250. doi: 10.1186/1475-2875-12-250. PubMed PMID: 23866736; PubMed Central PMCID: PMC3728046.

14. Schramm B, Valeh P, Baudin E, Mazinda CS, Smith R, Pinoges L, et al. Efficacy of artesunate-amodiaquine and artemether-lumefantrine fixed-dose combinations for the treatment of uncomplicated Plasmodium falciparum malaria among children aged six to 59 months in Nimba County, Liberia: an open-label randomized non-inferiority trial. Malar J. 2013;12:251. doi: 10.1186/1475-2875-12-251. PubMed PMID: 23866774; PubMed Central PMCID: PMC3728070.

15. Mayxay M, Khanthavong M, Lindegardh N, Keola S, Barends M, Pongvongsa T, et al. Randomized comparison of chloroquine plus sulfadoxine-pyrimethamine versus artesunate plus mefloquine versus artemether-lumefantrine in the treatment of uncomplicated falciparum malaria in the Lao People's Democratic Republic. Clin Infect Dis. 2004;39(8):1139-47. doi: 10.1086/424512. PubMed PMID: 15486837.

16. Faucher JF, Aubouy A, Adeothy A, Cottrell G, Doritchamou J, Gourmel B, et al. Comparison of sulfadoxine-pyrimethamine, unsupervised artemether-lumefantrine, and unsupervised artesunate-amodiaquine fixed-dose formulation for uncomplicated plasmodium falciparum malaria in Benin: a randomized effectiveness noninferiority trial. The Journal of infectious diseases. 2009;200(1):57-65. doi: 10.1086/599378. PubMed PMID: 19469703.

17. Tarning J, McGready R, Lindegardh N, Ashley EA, Pimanpanarak M, Kamanikom B, et al. Population pharmacokinetics of lumefantrine in pregnant women treated with artemether-lumefantrine for uncomplicated *Plasmodium falciparum* malaria. Antimicrobial agents and chemotherapy. 2009;53(9):3837-46. PubMed PMID: 19564366.

18. Vugt MV, Wilairatana P, Gemperli B, Gathmann I, Phaipun L, Brockman A, et al. Efficacy of six doses of artemether-lumefantrine (benflumetol) in multidrug-resistant Plasmodium falciparum malaria. The American journal of tropical medicine and hygiene. 1999;60(6):936-42. PubMed PMID: 10403324.

19. Ezzet F, Mull R, Karbwang J. Population pharmacokinetics and therapeutic response of CGP 56697 (artemether + benflumetol) in malaria patients. British journal of clinical pharmacology. 1998;46(6):553-61. PubMed PMID: 9862244.

20. van Vugt M, Looareesuwan S, Wilairatana P, McGready R, Villegas L, Gathmann I, et al. Artemether-lumefantrine for the treatment of multidrug-resistant falciparum malaria. Transactions of the Royal Society of Tropical Medicine and Hygiene. 2000;94(5):545-8. PubMed PMID: 11132386.

21. Denis MB, Tsuyuoka R, Lim P, Lindegardh N, Yi P, Top SN, et al. Efficacy of artemether-lumefantrine for the treatment of uncomplicated falciparum malaria in northwest Cambodia. Trop Med Int Health. 2006;11(12):1800-7. doi: 10.1111/j.1365-3156.2006.01739.x. PubMed PMID: 17176344.

22. Ashley EA, Stepniewska K, Lindegardh N, McGready R, Annerberg A, Hutagalung R, et al. Pharmacokinetic study of artemether-lumefantrine given once daily for the treatment of uncomplicated multidrug-resistant falciparum malaria. Trop Med Int Health. 2007;12(2):201-8. PubMed PMID: 17300626.

23. McGready R, Stepniewska K, Lindegardh N, Ashley EA, La Y, Singhasivanon P, et al. The pharmacokinetics of artemether and lumefantrine in pregnant women with uncomplicated falciparum malaria. European journal of clinical pharmacology. 2006;62(12):1021-31. PubMed PMID: 17053895.

24. van Vugt M, Brockman A, Gemperli B, Luxemburger C, Gathmann I, Royce C, et al. Randomized comparison of artemether-benflumetol and artesunate-mefloquine in treatment of multidrug-resistant falciparum malaria. Antimicrobial agents and chemotherapy. 1998;42(1):135-9. PubMed PMID: 9449273; PubMed Central PMCID: PMC105468.

25. Lefevre G, Looareesuwan S, Treeprasertsuk S, Krudsood S, Silachamroon U, Gathmann I, et al. A clinical and pharmacokinetic trial of six doses of artemether-lumefantrine for multidrug-resistant Plasmodium falciparum malaria in Thailand. The American journal of tropical medicine and hygiene. 2001;64(5-6):247-56. PubMed PMID: 11463111.
