# Supplementary material for: Artemether-lumefantrine dosing for malaria treatment in young children and pregnant women: A pharmacokinetic-pharmacodynamic meta-analysis
Source: PLoS Med. 2018 Jun 12;15(6):e1002579. doi: 10.1371/journal.pmed.1002579 (PMC5997317; doi:10.1371/journal.pmed.1002579)
Supplement: S2 Table — (DOCX) [file pmed.1002579.s008.docx]

| **Table 2:** demographic summary of post-2013 studies meeting the original inclusion criteria. | | | | | | | | | | | | | | | | | |
| --- | --- | --- | --- | --- | --- | --- | --- | --- | --- | --- | --- | --- | --- | --- | --- | --- | --- |
| Reference | Study site | Study size | Dose regimens | Dose | Sampling matrices | Sample size LF | Samples/patient LF | Sample size DLF | Samples/patient DLF | Male/female | Age (year) | Bodyweight (kg) | Z-score (weight for age) | Parasitaemia | Pregnant women (%) | Estimated gestational age (weeks) |  |
| [[1](#_ENREF_1)] | Uganda | Intensive sampling  145  54 HIV uninfected  91 HIV infected | 6 doses over 3 days | HIV infected  12.3 (8.05-16.1) mg/kg | Venous plasma | 2444 | 13 | - | - | - | HIV uninfected  3.5 (1.1-7.9) | 13.9 (9.8-27) | - | 161,189 (12,042-21,763) | 0 | - |  |
|  |  |  |  |  |  |  |  |  |  |  | EFV  5.6 (3.1-8.6) | 17.6 (11.4-25.1) | - | 11,671 (6,389-21,321) |  |  |  |
|  |  | Spars sampling  225  134 HIV uninfected  91 HIV infected |  | HIV uninfected  10.9 (8.05-16.0) mg/kg | Capillary plasma | 675 | 3 |  |  |  | LPV  4.6 (1.4-8.0) | 15.1 (7.7-23.7) | - | 6,917 (3,839-12,463) |  |  |  |
|  |  |  |  |  |  |  |  |  |  |  | NVP  4.6 (1.4-8.0) | 16.3 (8.5-30) | - | 10,568 (5,746-19,435) |  |  |  |
| [[2](#_ENREF_2)] | Malawi  Uganda | NVP  16 | 6 doses over 3 days | <15kg: 120 mg  15-24.9 kg: 240 mg  25-34.9 kg:  360 mg  > 35 kg: 480 mg | Venous blood | 152 | 8 | - |  | NVP  63/38 | NVP  7.1 (4.1. – 11.3) | NVP  17.9  (14.0 – 29.3) | - | <200,000u/L | 0 | - |  |
|  |  | ART naïve  3 |  |  |  |  |  |  |  | ART naïve  33/67 | ART naïve  8.3 (3.6 – 10.0) | 19.7 (16.1 – 27.0) | - |  |  |  |  |
| [[3](#_ENREF_3)] | Tanzania | 55 | 6 doses over 3 days | 480 mg | Venous blood | 165 | 5 | 33 | 165 | 0/55 | Pregnant: 25 (18-41) Non-pregnant: 21.5 (18-35) | Pregnant: 52 (40-80)  Non-pregnant:  48.5 (41-79) | - | Pregnant: 25,280 (560-198,080)  Non-pregnant: 22,280 (560–195,680) | 60 | 27 (14-37) |  |
| [[4](#_ENREF_4)] | Uganda | 60 | 6 doses over 3 days | 480 mg | Capillary blood and venous plasma | 390 | 13 | - | - | 11/49 | Pregnant: 25 (18-39)  Non-pregnant:  24 (16-68) | Pregnant: 59.4 (44.5-81.1)  Non-pregnant:  55.7 (38.0–68.4) | - | Pregnant:  13,227 (7,728-22,639)  Non-pregnant:  597 (261–1,371) | 100 | 28 (14-34) |  |
| [[5](#_ENREF_5)] | Tanzania | 92 | 6 doses over 3 days | 480 mg | Venous blood | 92 | 1 | 0 | 0 | 0/92 | 23 (15-41) | - | - | 2,700 (400-72,500) | 100 | 2^nd^ & 3^rd^ trimesters |  |
| [[6](#_ENREF_6)] | Uganda | 101 | 6 doses over 3 days | <=12 months: 14.1 (10.9-19.7) mg/kg  12-18 months: 13.3 (9.23-19.4) mg/kg  >=18 months: 12.0 (9.02-15.0) mg/kg  UWFA received a higher median mg/kg dose 90.0 mg/kg vs 77.4 mg/kg | Capillary blood | 806 | 5 | - | - | 48/53 | 1.2 (0.55-1.85) | 9.1 (6.1-13.0) | - | 15,568 (128-159,393) | 0 | - |  |
| [[7](#_ENREF_7)] | Mali & Niger | 131 (SAM)  132 (non-SAM) | 6 doses over 3 days | 120 mg | Capillary blood | 655  660 | 5 | - | - | 66/65  122/144 | 16.9 (SD: 7.7)  28.2 (SD: 13.0) years | 6.9 (1.1)  10.6 (2.5) | -3.39 (0.58)  -1.41 (0.93) | 10880 | 0 | - |  |

EFV: efavirenz, LPV: lopinavir, NVP: nevirapine, ART: antiretroviral therapy and SAM: Severe acute malnutrition. Reported as median (range) unless otherwise specified.

## References

1. Parikh S, Kajubi R, Huang L, Ssebuliba J, Kiconco S, Gao Q, et al. Antiretroviral Choice for HIV Impacts Antimalarial Exposure and Treatment Outcomes in Ugandan Children. Clin Infect Dis. 2016;63(3):414-22. doi: 10.1093/cid/ciw291. PubMed PMID: 27143666; PubMed Central PMCID: PMCPMC4946019.

2. Huang L, Carey V, Lindsey JC, Marzan F, Gingrich D, Graham B, et al. Concomitant nevirapine impacts pharmacokinetic exposure to the antimalarial artemether-lumefantrine in African children. PloS one. 2017;12(10):e0186589. doi: 10.1371/journal.pone.0186589. PubMed PMID: 29065172; PubMed Central PMCID: PMCPMC5655345.

3. Mosha D, Guidi M, Mwingira F, Abdulla S, Mercier T, Decosterd LA, et al. Population pharmacokinetics and clinical response for artemether-lumefantrine in pregnant and nonpregnant women with uncomplicated Plasmodium falciparum malaria in Tanzania. Antimicrobial agents and chemotherapy. 2014;58(8):4583-92. doi: 10.1128/AAC.02595-14. PubMed PMID: 24867986; PubMed Central PMCID: PMC4136066.

4. Nyunt MM, Nguyen VK, Kajubi R, Huang L, Ssebuliba J, Kiconco S, et al. Artemether-Lumefantrine Pharmacokinetics and Clinical Response Are Minimally Altered in Pregnant Ugandan Women Treated for Uncomplicated Falciparum Malaria. Antimicrobial agents and chemotherapy. 2015;60(3):1274-82. doi: 10.1128/AAC.01605-15. PubMed PMID: 26666942; PubMed Central PMCID: PMCPMC4775973.

5. Mutagonda RF, Kamuhabwa AAR, Minzi OMS, Massawe SN, Asghar M, Homann MV, et al. Effect of pharmacogenetics on plasma lumefantrine pharmacokinetics and malaria treatment outcome in pregnant women. Malar J. 2017;16(1):267. doi: 10.1186/s12936-017-1914-9. PubMed PMID: 28673292; PubMed Central PMCID: PMCPMC5496343.

6. Tchaparian E, Sambol NC, Arinaitwe E, McCormack SA, Bigira V, Wanzira H, et al. Population Pharmacokinetics and Pharmacodynamics of Lumefantrine in Young Ugandan Children Treated With Artemether-Lumefantrine for Uncomplicated Malaria. The Journal of infectious diseases. 2016;214(8):1243-51. doi: 10.1093/infdis/jiw338. PubMed PMID: 27471317; PubMed Central PMCID: PMCPMC5034953.

7. Denoeud-Ndam L, Dicko A, Baudin E, Guindo O, Grandesso F, Diawara H, et al. Efficacy of artemether-lumefantrine in relation to drug exposure in children with and without severe acute malnutrition: an open comparative intervention study in Mali and Niger. BMC Med. 2016;14(1):167. doi: 10.1186/s12916-016-0716-1. PubMed PMID: 27776521; PubMed Central PMCID: PMCPMC5079061.
