# Supplementary material for: Artemether-lumefantrine dosing for malaria treatment in young children and pregnant women: A pharmacokinetic-pharmacodynamic meta-analysis
Source: PLoS Med. 2018 Jun 12;15(6):e1002579. doi: 10.1371/journal.pmed.1002579 (PMC5997317; doi:10.1371/journal.pmed.1002579)
Supplement: S1 Text — (DOCX) [file pmed.1002579.s009.docx]

## Supplementary methods

### Lumefantrine pharmacokinetic model

A variety of distribution models (one-, two-, and three-compartment distribution at *P*<*0.001*), absorption models (first-order, first-order with lag-time, and transit-absorption at *P*<*0.001*) and residual error models (additive, proportional, and combined additive and proportional error models) were evaluated. A conservative cut-off significance value of 0.001 (*P*<*0.001*) was used for comparison of hierarchical distribution and absorption models to ensure that only highly significant improvements in model fit were propagated during the model building phase. Random variability between patients was coded log-normally (Equation 1):

$P_{i}=\theta\times e^{\eta}$ Equation 1

P_i_ in Equation 1 represents the individual parameter estimate, θ represents the typical population estimate and η represents normally distributed between patient variability, subsequently scaled log-normally. This variability structure enabled estimation of variability in densely and sparsely sampled studies simultaneously. Between doses (inter-occasion) variability was not evaluated in this study due to the sparseness of data in some studies which would have resulted in absence of date for some dose occasions. Baseline concentrations were estimated for baseline samples higher than the lower limit of quantification by estimating the amount of lumefantrine and/or desbutyl-lumefantrine in the respective sampling compartment at baseline. Relative bioavailability (implemented as fixed to unity for the population) was also embedded in the model to allow inter-individual variability in the absorption of lumefantrine. The First Order Conditional Estimation (FOCE) method was used for the pharmacokinetic models. The FOCE method is a gradient based estimation method in the software package NONMEM. NONMEM is the gold standard for population analyses in clinical pharmacology and the FOCE estimation method tends to be more robust as compared to expectation maximization estimation methods [[1](#_ENREF_1)]. Different disposition models were evaluated whilst accounting for possible model misspecification caused by data below the limit of quantification [[2](#_ENREF_2)]. For this purpose the Laplacian estimation method was used which maximises the probability that observed concentration data below the limit of quantification is also estimated to be below the limit of quantification (i.e. M3-method) [[3](#_ENREF_3)]. However, no model misspecification was found on account of below limit of quantification data and therefore the FOCE method was used during the rest of the model building process. Physiological plausibility, goodness-of-fit diagnostics (observations vs population predictions and individual predictions, and conditionally weighted residuals vs time and population predictions) and the objective function value (-2LL: computed as proportional to minus twice the log likelihood of the data) were used to evaluate competing models. A significant (*P<0.05*) improvement, after the introduction of one new parameter (one degree of freedom), was concluded if the -2LL dropped with 3.84 points or more. For further evaluations of competing models the Akaike information criterion was used (calculated as: -2LL + 2 × n_parameters_).

Covariates were carried forward if statistical improvement was realised using a stepwise covariate modelling approach. A less stringent p-value (*p<0.05*) was used during the forward step of the stepwise covariate model building procedure to allow inclusion of all potentially relevant covariates. Covariates were eliminated using a more conservative p-value *(P<0.001*) in the backward step of the stepwise covariate modelling procedure in order to allow only highly influential covariates to be retained in the final model. For pregnancy, an additional criterion was that the covariate had to be significant when evaluated both on all data and on matched data only (study population and matching random control population). Bodyweight was evaluated using a power relationship on all clearance and volume parameters with coefficients either fixed to 0.75 and 1, respectively, or estimated. Disease effect was evaluated using baseline parasitaemia as continuous covariate, pregnancy was evaluated using a categorical covariate and estimated gestational age as a continuous covariate. Factors potentially impacting lumefantrine absorption such as dosage (mg/kg), dose per occasion (mg), total daily dose (mg), total dose (mg) and age for weight z-score were evaluated on bioavailability using a saturation model (Equation 2).

 Equation 2

Covariate_50_ in the saturation model (equation 2) represents an estimated value, which realises half of the maximum covariate effect. This logistic-type covariate effect was used to describe saturation of bioavailability in order to constrain the covariate effect on relative bioavailability to be between zero and one.

Admission axillary body temperature and admission haemoglobin was studied in a previous analysis on lumefantrine day 7 concentrations [[4](#_ENREF_4)]. However, these covariates were not available for more than 20% of the patients in the current population, and admission body temperature and haemoglobin was therefore not considered in the formal covariate analysis. Instead, these covariates were evaluated in a separate sub-group analysis including only patients in whom these covariates were available to enable a comparison with previous studies.

Models were validated using prediction-corrected visual predictive checks, bootstraps and external validation. For the prediction-corrected visual predictive check, 2,000 new studies were generated *in-silico* and 90% confidence intervals of the simulated 5^th^, 50^th^ and 95^th^ percentiles were overlaid with the observed 5^th^, 50^th^ and 95^th^ percentiles of the data. Model misspecification, in that predicted confidence intervals for one or more of the percentiles did not overlap with the corresponding observed percentiles, resulted in further development of the model through re-parameterisation. For the bootstrap, 1,000 new datasets were re-sampled with replacement from the original dataset and subsequently refitted with the final model. Relative standard errors were reported as 100 × standard deviation/mean and 95% confidence intervals as the 2.5th and 97.5thpercentiles of the bootstrap results. External validation was conducted using all data not used for model building.

Proportional conversion factors at residual error level were estimated for venous blood, capillary blood, capillary plasma, dispersible tablets and crushed tablets. The conversion factors were estimated with all other parameters in the model fixed to the best performing venous plasma lumefantrine model estimates. The conversion factor consequently enabled proportional scaling of predicted concentrations at a population level whilst population and between subject variability estimates remained unaffected. Prediction-corrected visual predictive checks were subsequently performed to evaluate the predictive performance on the external validation data comprising patients contributing < 2 venous plasma samples.

### Time-to-event model

Interval censoring was implemented for recrudescent malaria infections. The start of the interval was set to the time of the last negative malaria visit. When there was no information available for recrudescent malaria patients regarding their last negative visit, the start of the interval was arbitrary set to one week prior to the recrudescent infection.

Recrudescent malaria was evaluated using a baseline hazard (Equation 3), Weibull hazard (Equation 4) and gompertz (Equation 5) hazard model.

$h(t)=\lambda$ Equation 3

$h(t)=\alpha^{2}\times\lambda\times t^{\alpha-1}$ Equation 4

$h(t)=\lambda\times e^{-t\times\frac{ln(2)}{t_{1/2}}}$ Equation 5

Equation 3-5 represent hazard over time (h(t)), baseline hazard (λ), time to reach half-maximum hazard (t_1/2_) and shape parameter (α). Physiological plausibility, prediction corrected visual predictive checks and the objective function value were used to evaluate competing models. The lumefantrine drug effect was subsequently studied using an E_MAX_ model and a sigmoidal E_MAX_ model. Stepwise covariate modelling on pharmacodynamic parameters for recrudescent malaria (i.e. age, bodyweight, weight for age z-scores, pregnancy, estimated gestational age and admission parasitaemia) was conducted using a forward (*P*<*0.05*) and backward elimination approach (*p<0.001*).

### *In-silico* dose optimisations

Parasite biomass at baseline was described using baseline bodyweight and pregnancy in a linear regression model. The linear regression model was subsequently used to account for correlation structures in covariates whilst creating virtual patient populations in the simulation scenarios. *In-silico* dose optimisations were conducted using 2,000 simulations of the day 7 lumefantrine concentration, total exposure, and maximum lumefantrine concentration. Uncertainty in parameter estimates was not accounted for considering that dose optimisations aimed to establish similar mean exposures in vulnerable populations (i.e. children and pregnant women) as compared to non-pregnant adults (i.e. additional variability introduced by uncertainty should not introduce bias and would be centred around the mean).

### Simultaneous lumefantrine/desbutyl-lumefantrine pharmacokinetic model

A variety of distribution models (one-, two-, and three-compartment distribution) and residual error models (additive, proportional, and combined additive and proportional error models) were evaluated for desbutyl-lumefantrine using the best performing lumefantrine model. Subsequently, an identical procedure for covariate modelling was applied to desbutyl-lumefantrine parameters as described for the lumefantrine. Validation of the simultaneous lumefantrine/desbutyl-lumefantrine model was also identical to the procedures followed for the lumefantrine.

## References

1. Pradhan S, Song B, Lee J, Chae JW, Kim KI, Back HM, et al. Performance comparison of first-order conditional estimation with interaction and Bayesian estimation methods for estimating the population parameters and its distribution from data sets with a low number of subjects. BMC Med Res Methodol. 2017;17(1):154. doi: 10.1186/s12874-017-0427-0. PubMed PMID: 29191177; PubMed Central PMCID: PMCPMC5709938.

2. Hendriksen IC, Mtove G, Kent A, Gesase S, Reyburn H, Lemnge MM, et al. Population pharmacokinetics of intramuscular artesunate in African children with severe malaria: implications for a practical dosing regimen. Clin Pharmacol Ther. 2013;93(5):443-50. doi: 10.1038/clpt.2013.26. PubMed PMID: 23511715; PubMed Central PMCID: PMCPMC3630454.

3. Ahn JE, Karlsson MO, Dunne A, Ludden TM. Likelihood based approaches to handling data below the quantification limit using NONMEM VI. J Pharmacokinet Pharmacodyn. 2008;35(4):401-21. doi: 10.1007/s10928-008-9094-4. PubMed PMID: 18686017.

4. WorldWide Antimalarial Resistance Network Lumefantrine PKPDSG. Artemether-lumefantrine treatment of uncomplicated Plasmodium falciparum malaria: a systematic review and meta-analysis of day 7 lumefantrine concentrations and therapeutic response using individual patient data. BMC Med. 2015;13:227. doi: 10.1186/s12916-015-0456-7. PubMed PMID: 26381375; PubMed Central PMCID: PMCPMC4574542.
