# Supplementary material for: Artemether-lumefantrine dosing for malaria treatment in young children and pregnant women: A pharmacokinetic-pharmacodynamic meta-analysis
Source: PLoS Med. 2018 Jun 12;15(6):e1002579. doi: 10.1371/journal.pmed.1002579 (PMC5997317; doi:10.1371/journal.pmed.1002579)
Supplement: S2 Text — (DOCX) [file pmed.1002579.s010.docx]

## Supplementary results

### Lumefantrine pharmacokinetic model

The lumefantrine venous plasma concentration-time data (mostly distributed between 0 and 300 hours) in the current pooled dataset was best described by a two-compartment disposition model. Previously published lumefantrine models, in which patients were sampled for 14 days (336 hours) after starting treatment [[1-3](#_ENREF_1)], have also used two-compartment distribution models. One study in Papua New Guinean children used a three-compartment disposition model to describe lumefantrine distribution pharmacokinetics after sampling for more than 600 hours [[4](#_ENREF_4)]. Employing a second peripheral compartment suggests a longer terminal elimination half-life. This could contribute to a prolonged post-treatment prophylactic period (i.e. when lumefantrine concentrations remain above the minimum effective concentrations). Adding a second peripheral compartment in the current model did not improve the model fit, although the majority of data were obtained before 300 hours.

A first-order absorption model described the lumefantrine absorption characteristics adequately (Figure 2). First-order absorption with lag-time did not converge but a transit absorption model (6 transit compartments) with estimated transit and absorption rate constants converged and resulted in a significant improvement of the model fit compared to a first-order absorption model (*p < 0.001*; ∆-2LL = -615; ∆AIC = -615). However, simulation-based diagnostics (n = 2,000) demonstrated that the transit absorption model resulted in clear model misspecification during the absorption phase (< 8 hours after dose), indicated by an under-prediction of the simulated 5^th^ percentile of data compared to the observed 5^th^ percentile. There was no improvement in the simulation-based diagnostics after 8 hours post-administration when compared to a first-order absorption model. Therefore, a simpler first-order absorption model was implemented in the final pharmacokinetic model.

***Disease and dosage related covariate models***

Apart from the pre-treatment parasite count covariate on relative bioavailability there was also a statistically significant relationship (exponential) between pre-treatment parasitaemia and peripheral lumefantrine distribution volume resulting in increased peripheral distribution volume with increasing pre-treatment parasite counts (exponent of 0.273; *p < 0.001*; ∆-2LL = -22.5; ∆AIC = -20.5). However, when the model was run on non-pregnant adult data only, pre-treatment parasitaemia was a significant covariate on relative bioavailability only. This suggested that the correlation between baseline parasitaemia and lumefantrine peripheral distribution volume was confounded by age and/or weight and it was therefore not included in the final model.

***Children***

Scaling of clearance and volume parameters using body weight as an allometric function was used in this model to describe changes in body size, as has been reported previously in lumefantrine population pharmacokinetic models in children and pregnant women [[1](#_ENREF_1), [4](#_ENREF_4)]. Traditional fixed allometry was considered superior to estimated power coefficients since the latter resulted in unstable parameter estimates, probably because of over parameterisation.

***Pregnant women***

Pregnant women in their second and third pregnancy trimester displayed a substantially higher lumefantrine absorption rate when compared to non-pregnant adults. The quicker, but not increased, absorption translated in altered distribution pharmacokinetics resulting in lower lumefantrine concentrations at day 7, a drug exposure surrogate that has previously been associated with therapeutic responses [[5-7](#_ENREF_5)]. This was in agreement with previously published population pharmacokinetic analyses also showing lower predicted lumefantrine day 7 concentrations in pregnant women compared to non-pregnant adults [[1](#_ENREF_1), [2](#_ENREF_2), [8](#_ENREF_8)]. This underexposure was previously ascribed to a pregnancy effect on inter-compartmental clearance or an estimated gestational age effect on apparent central distribution volume [[1](#_ENREF_1), [2](#_ENREF_2)]. However, our analysis suggests that the lower lumefantrine exposure in pregnancy results from altered absorption and distribution related parameters.

***Matrix and formulation effects***

All model parameters were fixed and a correction factor, aimed at accounting for differences in sampling matrix and formulation effects, was estimated. Subsequently, the model was validated using a prediction corrected visual predictive check. Over-prediction of the variability of venous blood (Appendix figure 2D) and capillary plasma (Appendix figure 2F) concentrations, used for external validation, could be explained by the differences in residual variability in the data. The model was developed using a variety of different studies resulting in a large residual variability due to their use of different study methodology and populations. However, in the observed sparse venous blood and capillary plasma external validation data much less variability was present since all lumefantrine concentration samples were from one study.

### *In-silico* dose optimisation

A linear regression model for baseline parasite biomass as dependent variable and baseline bodyweight ([estimate; standard error] -0.011410; 0.001184), pregnancy status (-0.939481; 0.126401) with an intercept (4.366136; 0.048146) as predictors was highly significant (all p-values < 2e^-13^).

### Lumefantrine/desbutyl-lumefantrine pharmacokinetic model

A two-compartment distribution model for desbutyl-lumefantrine pharmacokinetics was in line with a previously published lumefantrine desbutyl-lumefantrine pharmacokinetic model in Papua New Guinean children [[4](#_ENREF_4)]. Bodyweight, implemented using a power relationship on clearance and volume parameters explained the changes in body size as seen in a previous publication [[4](#_ENREF_4)].

Including the effect of pregnancy (as a categorical covariate) on apparent desbutyl-lumefantrine peripheral distribution volume resulted in a significant improvement in model fit (coefficient -75.9%; p < 0.001; ∆-2LL = -24.5; ∆AIC = -22.5) when only pregnant and matching non-pregnant women were studied. However, when all data were analysed the model was unstable (i.e. ∆-2LL = ±100 with different sets of initial parameter estimates). Therefore, pregnancy implemented as a categorical covariate on apparent desbutyl-lumefantrine peripheral distribution volume was not included. Estimated gestational age as an exponential continuous covariate on apparent desbutyl-lumefantrine peripheral distribution volume resulted in a significant improvement of the model fit (exponent of -0.045; p < 0.001; ∆-2LL = -21.3; ∆AIC = -19.3) when all data were studied. However, this model was also unstable (i.e. zero-gradients) when only pregnant and matched non-pregnant women were studied. Therefore, estimated gestational age implemented as a continuous covariate on apparent desbutyl-lumefantrine peripheral distribution volume, in pregnant women during their second and third trimester, was not included. This lack of robustness might be explained by the small sample size in pregnant patients (14 patients with 15 samples per patient [3-16]) and matching non-pregnant patients (10 patients with 3 samples per patient [3-4]).

## References

1. Kloprogge F, Piola P, Dhorda M, Muwanga S, Turyakira E, Apinan S, et al. Population Pharmacokinetics of Lumefantrine in Pregnant and Nonpregnant Women With Uncomplicated Plasmodium falciparum Malaria in Uganda. CPT: pharmacometrics & systems pharmacology. 2013;2:e83. Epub 2013/11/15. doi: 10.1038/psp.2013.59. PubMed PMID: 24226803.

2. Tarning J, McGready R, Lindegardh N, Ashley EA, Pimanpanarak M, Kamanikom B, et al. Population pharmacokinetics of lumefantrine in pregnant women treated with artemether-lumefantrine for uncomplicated *Plasmodium falciparum* malaria. Antimicrobial agents and chemotherapy. 2009;53(9):3837-46. PubMed PMID: 19564366.

3. Tchaparian E, Sambol NC, Arinaitwe E, McCormack SA, Bigira V, Wanzira H, et al. Population Pharmacokinetics and Pharmacodynamics of Lumefantrine in Young Ugandan Children Treated With Artemether-Lumefantrine for Uncomplicated Malaria. The Journal of infectious diseases. 2016;214(8):1243-51. doi: 10.1093/infdis/jiw338. PubMed PMID: 27471317; PubMed Central PMCID: PMCPMC5034953.

4. Salman S, Page-Sharp M, Griffin S, Kose K, Siba PM, Ilett KF, et al. Population pharmacokinetics of artemether, lumefantrine, and their respective metabolites in Papua New Guinean children with uncomplicated malaria. Antimicrobial agents and chemotherapy. 2011;55(11):5306-13. Epub 2011/08/31. doi: 10.1128/AAC.05136-11. PubMed PMID: 21876056; PubMed Central PMCID: PMC3194999.

5. Price RN, Uhlemann AC, van Vugt M, Brockman A, Hutagalung R, Nair S, et al. Molecular and pharmacological determinants of the therapeutic response to artemether-lumefantrine in multidrug-resistant Plasmodium falciparum malaria. Clin Infect Dis. 2006;42(11):1570-7. PubMed PMID: 16652314.

6. Ezzet F, Mull R, Karbwang J. Population pharmacokinetics and therapeutic response of CGP 56697 (artemether + benflumetol) in malaria patients. British journal of clinical pharmacology. 1998;46(6):553-61. PubMed PMID: 9862244.

7. WorldWide Antimalarial Resistance Network Lumefantrine PKPDSG. Artemether-lumefantrine treatment of uncomplicated Plasmodium falciparum malaria: a systematic review and meta-analysis of day 7 lumefantrine concentrations and therapeutic response using individual patient data. BMC Med. 2015;13:227. doi: 10.1186/s12916-015-0456-7. PubMed PMID: 26381375; PubMed Central PMCID: PMCPMC4574542.

8. McGready R, Stepniewska K, Lindegardh N, Ashley EA, La Y, Singhasivanon P, et al. The pharmacokinetics of artemether and lumefantrine in pregnant women with uncomplicated falciparum malaria. European journal of clinical pharmacology. 2006;62(12):1021-31. PubMed PMID: 17053895.
